# Engineered biosynthesis of milbemycins in the avermectin high-producing strain *Streptomyces avermitilis*

**DOI:** 10.1186/s12934-017-0626-8

**Published:** 2017-01-17

**Authors:** Myoun-Su Kim, Wan-Je Cho, Myoung Chong Song, Seong-Whan Park, Kaeun Kim, Eunji Kim, Naryeong Lee, Sang-Jip Nam, Ki-Hoon Oh, Yeo Joon Yoon

**Affiliations:** 1 Department of Chemistry and Nano Science, Ewha Womans University, 52, Ewhayeodae-gil, Seoul, 03760 Republic of Korea; 2 Crop Protection R&D Center, FarmHannong Co., Ltd, 39-23, Dongan-ro 1113beon-gil, Yeonmu-eup, Nonsan-si, Chungcheongnam-do 33010 Republic of Korea; 3 Department of Bioinformatics, Bio Campus of Korea Polytechnic, 48, Dongan-ro 112-gil, Ganggyeong-eup, Nonsan-si, Chungcheongnam-do 32943 Republic of Korea

**Keywords:** Milbemycins, Avermectins, Biosynthesis, *Streptomyces avermitilis*

## Abstract

**Background:**

Milbemycins, produced from *Streptomyces hygroscopicus* subsp. *aureolacrimosus* and *Streptomyces bingchenggensis*, are 16-membered macrolides that share structural similarity with avermectin produced from *Streptomyces avermitilis*. Milbemycins possess strong acaricidal, insecticidal, and anthelmintic activities but low toxicity. Due to the high commercial value of the milbemycins and increasing resistance to the avermectins and their derivatives, it is imperative to develop an efficient combinatorial biosynthesis system exploiting an overproduction host strain to produce the milbemycins and novel analogs in large quantities.

**Results:**

The respective replacement of AveA1 and AveA3 (or module 7 in AveA3) of the avermectin polyketide synthase (PKS) in the avermectin high-producing strain *S. avermitilis* SA-01 with MilA1 and MilA3 (or module 7 in MilA3) of the milbemycin PKS resulted in the production of milbemycins A3, A4, and D in small amounts and their respective C5-*O*-methylated congener milbemycins B2, B3, and G as major products with total titers of approximately 292 mg/l. Subsequent inactivation of the C5-*O*-methyltransferase AveD led to a production of milbemycins A3/A4 (the main components of the commercial product milbemectin) in approximately 225 and 377 mg/l in the flask and 5 l fermenter culture, respectively, along with trace amounts of milbemycin D.

**Conclusions:**

We demonstrated that milbemycin biosynthesis can be engineered in the avermectin-producing *S. avermitilis* by combinatorial biosynthesis with only a slight decrease in its production level. Application of a similar strategy utilizing higher producing industrial strains will provide a more efficient combinatorial biosynthesis system based on *S. avermitilis* for further enhanced production of the milbemycins and their novel analogs with improved insecticidal potential.

**Electronic supplementary material:**

The online version of this article (doi:10.1186/s12934-017-0626-8) contains supplementary material, which is available to authorized users.

## Background

**Fig. 1:**
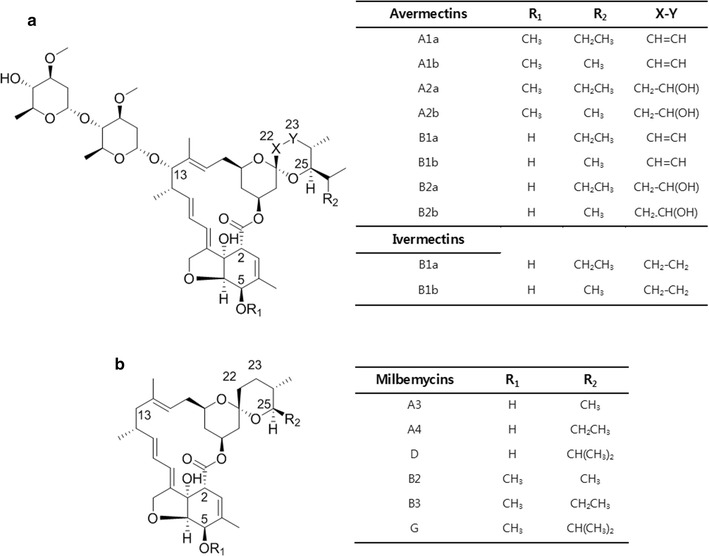
The structures of **a** Avermectins and ivermectins, **b** Milbemycins

The milbemycins and avermectins are structurally related 16-membered macrolides with excellent anthelmintic and insecticidal activities (Fig. 1). Avermectins, produced by *Streptomyces avermitilis*, are composed of a pentacyclic macrolactone and a disaccharide of methylated l-oleandrose [1, 2]. Avermectin B1 homologs which possess a C5-hydroxy group and a double bond between C22–C23 are the most potent congeners among the eight structurally related avermectin compounds [3]. Consequently, the semisynthetic 22,23-dihydroavermectin B1 (ivermectin) (Fig. 1) was developed and introduced to the market for use as an antiparasitic agent [4]. Furthermore, the increase of avermectin and ivermectin-resistant insects has led to the development of a series of avermectin analogs including selamectin, abamectin, emamectin, and doramectin [5]. The milbemycins were originally isolated from *Streptomyces hygroscopicus* subsp. *aureolacrimosus* [6] and recently from *Streptomyces bingchenggensis* [7, 8]. While the structures of milbemycins are similar to those of the avermectins, a bisoleandrosyl substituent appended to the C13 position of the avermectins’ macrolide ring does not exist in milbemycins. Only a single bond at the C22–C23 position is found in milbemycins, whereas the avermectins can possess a single or double bond at the same position. In addition, several different alkyl chains are attached at the C25 position of these compounds (Fig. 1). They possess broad acaricidal and insecticidal activities with relatively lower toxicity to non-target organisms [9, 10]. Milbemectin (a mixture of milbemycins A3 and A4) was first commercialized as an acaricide [11]. Subsequently, a series of milbemycins analogs, such as lepimectin (13-(α-methoxyiminophenylacetoxy)milbemycin) [12] and milbemycin oxime (5-oxime derivate of milbemycins A3/A4) [13], have been developed.

**Fig. 2:**
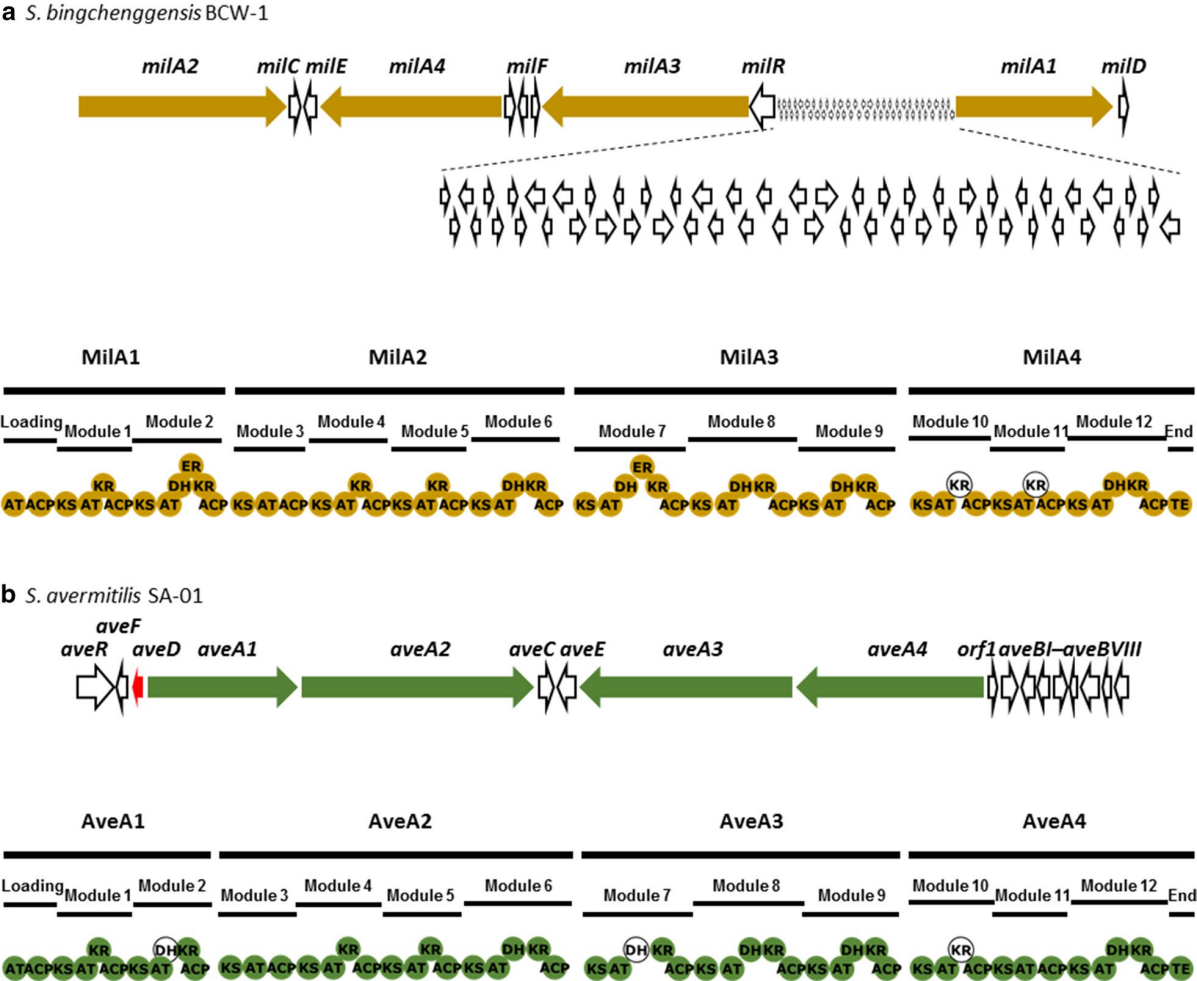
Gene and module organizations of avermectin and milbemycin biosynthetic gene clusters. **a** Milbemycin PKS in *S. bingchenggensis* BCW-1. The PKS genes (*milA1*, *milA2*, *milA3*, and *milA4*) for milbemycin biosynthesis were indicated with *yellow*. **b** Avermectin PKS in *S. avermitilis* SA-01. The PKS genes (*aveA1*, *aveA2*, *aveA3*, and *aveA4*) for the biosynthesis of the avermectin core skeleton were shown with *green*. The *aveD* gene that was engineered in this study was shown with *red*. *Empty circles* indicate nonfunctional domains

The biosynthetic gene clusters of the milbemycins in *Streptomyces nanchangensis* NS3226 (milbemycins α11, α13, α14, β1, and β9 are identical to meilingmycins A to E, respectively, produced by *S. nanchangensis*) [14] and *S. bingchenggensis* [15] (Fig. 2a) as well as the avermectins in *S. avermitilis* [16] (Fig. 2b) were characterized. The milbemycin polyketide synthase (PKS) consists of four giant multifunctional polypeptides (MilA1, MilA2, MilA3, and MilA4). Its modular composition and gene organization are similar to those of avermectin PKS which possesses one loading module and twelve extension modules. The difference lies in the fact that the *milA1* gene encoding the loading module and two extension modules is located 55- and 62-kb apart from the other three PKS genes in the milbemycin gene cluster of *S. nanchangensis* and *S. bingchenggensis*, respectively, and there are several significant differences between the two biosynthetic genes at the domain level which directs the structural diversity of these molecules (Fig. 2). Specifically, the starting unit preference of the acyltransferase (AT) domain of the loading module, which determines the C25 substituents of the compounds, is different. Incorporation of 2-methylbutyryl-CoA or isobutyryl-CoA as a starter unit results in an isobutyl or isopropyl side chain in avermectin “a” and “b” components, respectively. In contrast, a methyl, ethyl, or isopropyl side chain at the C25 position of milbemycin results from the incorporation of an acetyl-CoA, propionyl-CoA, or isobutyryl-CoA, respectively. In addition, the unsaturated bond at the C22–C23 position in avermectin “1” components or the C23-hydroxy group in avermectin “2” components results from a completely inactive dehydratase (DH) domain in module 2 of the avermectin PKS and the unique activity of AveC catalyzing the dehydration-coupled spirocyclization [17]. On the other hand, the C22–C23 saturated bond of milbemycin is formed by the action of a fully active DH domain and enoylreductase (ER) domain in module 2 of milbemycin PKS. Additionally, the AveC counterpart MeiC in the meilingmycin biosynthetic gene cluster has spirocyclase activity but lacks the optional dehydratase activity [17]. Moreover, the DH domain of module 7 in avermectin PKS is presumed to be nonfunctional and is responsible for C13-hydroxy group formation through which a bisoleandrosyl substituent is connected, but the active DH and ER domains of milbemycin PKS generate a fully saturated structure at the corresponding position of milbemycin (Fig. 2).

There has been great interest in improving the yield of milbemycins A3 and A4 due to their prominent activity and commercial value. A combined approach of classical mutagenesis by ultraviolet irradiation or *N*-methyl-*N*′-nitroso-*N*-nitrosoguanidine and rational screening led to the mutant strain *S. bingchenggensis* with 80% enhanced production of milbemycins A3 and A4 compared to the parent strain [18]. However, this traditional method required time-consuming and laborious work. A simple metabolic engineering strategy also increased milbemycin A3/A4 titers in *S. bingchenggensis*. Deletion of the *milD* gene encoding a C5-*O*-methyltransferase and the *nanLD* gene encoding the loading module of the PKS for nanchangmycin (another major polyketide co-produced by *S. bingchenggensis*) constructed a mutant strain that produces approximately 74% more milbemycins A3/A4 than the initial *S. bingchenggensis* strain [19]. Although the genome of *S. bingchenggensis* was reported [15], a limited understanding of this milbemycin-producing strain may delay further improvement of milbemycin A3/A4 titers by conventional metabolic engineering.

The use of previously optimized and well-characterized industrial overproduction strains as a heterologous host could be an efficient alternative for the enhanced production of natural products [20, 21]. Recently, replacement of *aveA1* encoding the loading module and first two extension modules with *milA1* from the *S. hygroscopicus* HS023 industrial strain in a *S. avermitilis* G8-17 industrial strain produced 3.4 g/l of 25-methyl-22,23-dihydroavermectin and 25-ethyl-22,23-dihydroavermectin which displayed enhanced acaricidal and nematicidal activities [22]. Similarly, the same avermectin analogs were obtained by replacement of the loading module and DH-KR (ketoreductase) domains of module 2 in AveA1 with the loading module and DH-ER-KR domains of module 2 in MilA1 from *S. bingchenggensis* in another industrial avermectin-producing strain *S. avermitilis* NA-108 with similar yields [23].

The structural similarity of avermectin and milbemycin motivated us to produce milbemycins through engineering of the avermectin biosynthetic pathway in an *S. avermitilis* strain producing high levels of avermectins. In this report, we show that the *S. avermitilis* possessing previously enhanced avermectin production ability could be exploited for the production of milbemycins by replacement of the avermectin AveA1 and AveA3 (or module 7) PKS with milbemycin MilA1 and MilA3 (or module 7) PKS, respectively. The subsequent inactivation of AveD, which is responsible for the post-PKS methylation of the C5-hydroxy group [24], led to the production of milbemycins A3/A4 in great quantities in the engineered *S. avermitilis* strain.

## Results

### Design strategy for the engineered biosynthesis of milbemycins in *S. avermitilis*

To enable the biosynthesis of milbemycins in *S. avermitilis,* the avermectin PKS should be engineered as follows: (1) the substrate specificity of the AT domain of the loading module in AveA1 PKS should be altered to change the C25 side chains of avermectin to those present in the milbemycins, (2) the inactive DH domain of module 2 in AveA1 should be engineered to be fully active DH-ER domains in order to introduce a single bond at the C22–C23 position, and (3) the nonfunctional DH domain of module 7 in AveA3 should be engineered to be completely functional DH-ER domains to change the C13-hydroxy group of avermectin to the saturated methylene of milbemycin, which also prevents the attachment of the bisoleandrosyl moiety to the C13-hydroxy group. In order to engineer the AT domain of the loading module and the reductive domains of module 2, both domain/module swapping and the replacement of the entire PKS subunit are possible. Therefore, in order to reduce the number of time-consuming double crossover processes, we decided to replace the AveA1 PKS on the chromosome in *S. avermitilis* SA-01, the previously optimized avermectin high-producing mutant strain, with MilA1 from *S. hygroscopicus* subsp. *aureolacrimosus* NRRL 5739 rather than individually replace the corresponding domain/modules for changing the structures at C25 and C22–C23 of avermectins. To introduce the desired change at C13 and because domain organizations of AveA3 and MilA3 containing modules 7, 8, 9 are identical except for module 7, we attempted both the replacement of the PKS subunit AveA3 and swapping module 7 to investigate which strategy would be better to maintain the functional integrity of the hybrid PKS and maximize the production of the milbemycins.

**Fig. 3:**
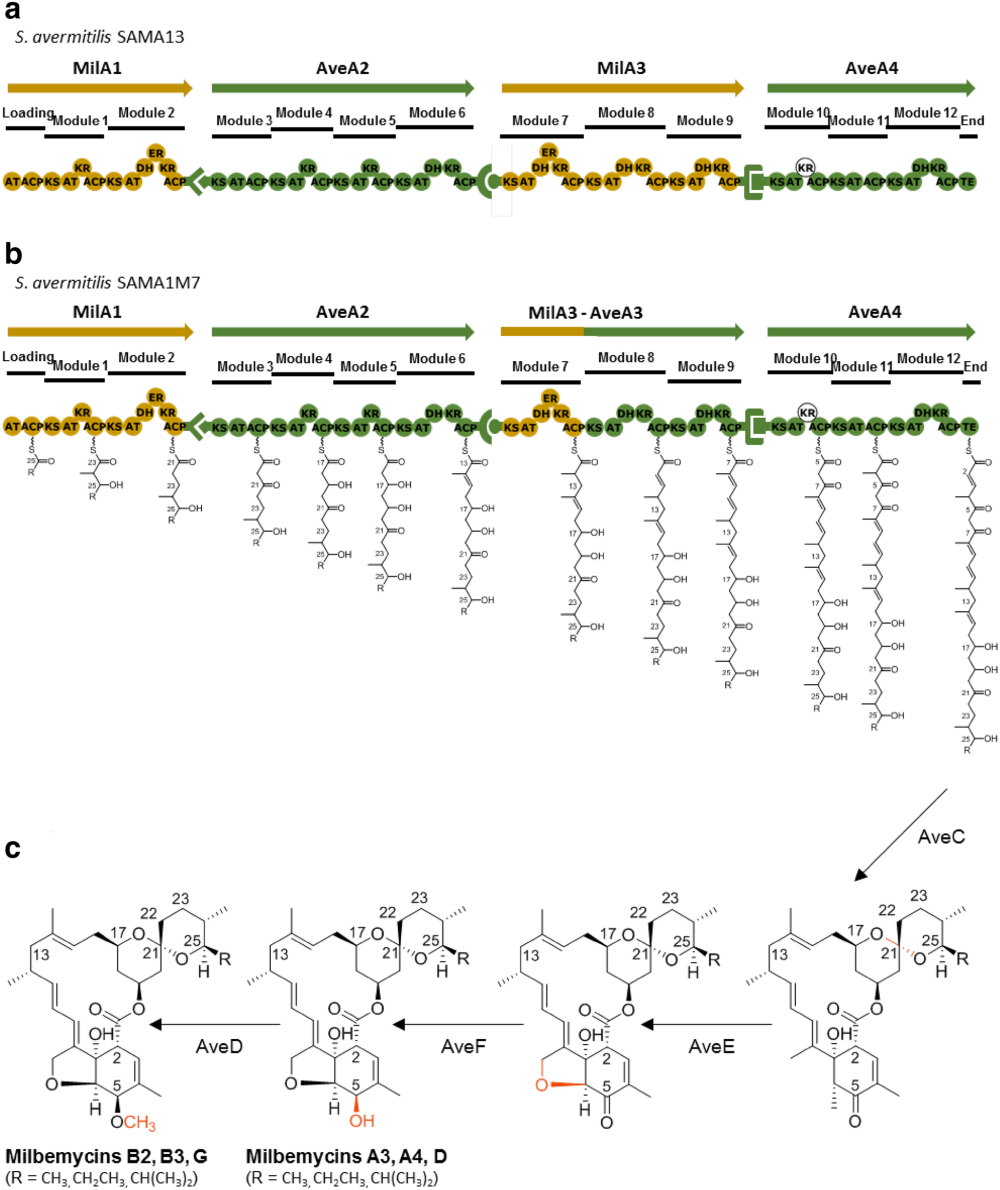
Engineered avermectin-milbemycin hybrid PKSs and predicted milbemycin biosynthetic pathways. **a** Modular organization in AveA1 and AveA3 replacement strain *S. avermitilis* SAMA13. **b** Modular organization in AveA1 and AveA3-module7 replacement strain *S. avermitilis* SAMA1M7. **c** Post-PKS tailoring pathway of desired milbemycins. The sequential tailoring steps were shown as *orange lines*

The docking domains at the termini of the PKS subunits are in part responsible for mediating the interaction of the corresponding subunits [25, 26]. To maintain the interaction between the substituted MilA1 and the downstream AveA2, the C-terminal docking domain of AveA1 was retained in the replaced MilA1. Similarly, the N- and C-terminal docking domains of AveA3 were maintained in the substituted MilA3 to keep the interactions of the replaced MilA3 with the upstream AveA2 and the downstream AveA4 (Fig. 3a). In the case of module 7 replacement, the N-terminal docking domain of AveA3 was also retained to maintain the subunit interactions between AveA2 and the module 7-replaced hybrid AveA3 (Fig. 3b). The boundaries of docking domains and the intermodular linker were chosen from the end of the highly conserved amino acid sequence of ACP (acyl carrier protein) domain in the upstream module to the start of the highly conserved sequence in the ketosynthase (KS) domain in the downstream module. The engineered restriction sites for modular fusion were designed not to change amino acid residues to minimize their adverse effects on the function of hybrid PKS and to keep its protein integrity (Additional file 1: Figure S1). The resulting *S. avermitilis* mutants in which AveA1 and AveA3 (or module 7 of AveA3) were replaced with MilA1 and MilA3 (or module 7 of MilA3), respectively, were expected to produce milbemycins through the post-PKS modification of the engineered polyketide chain by AveC (spirocyclase with optional dehydratase activity), AveE (cytochrome P450 involved in furan ring closure at C6–C8), AveF (dehydrogenase involved in C5-keto reduction), and AveD (C5-*O*-methyltransferase) of the host *S. avermitilis* strain (Fig. 3c).

### Construction of *aveA1* and *aveA3* (module 7) replacement mutant strains

**Fig. 4:**
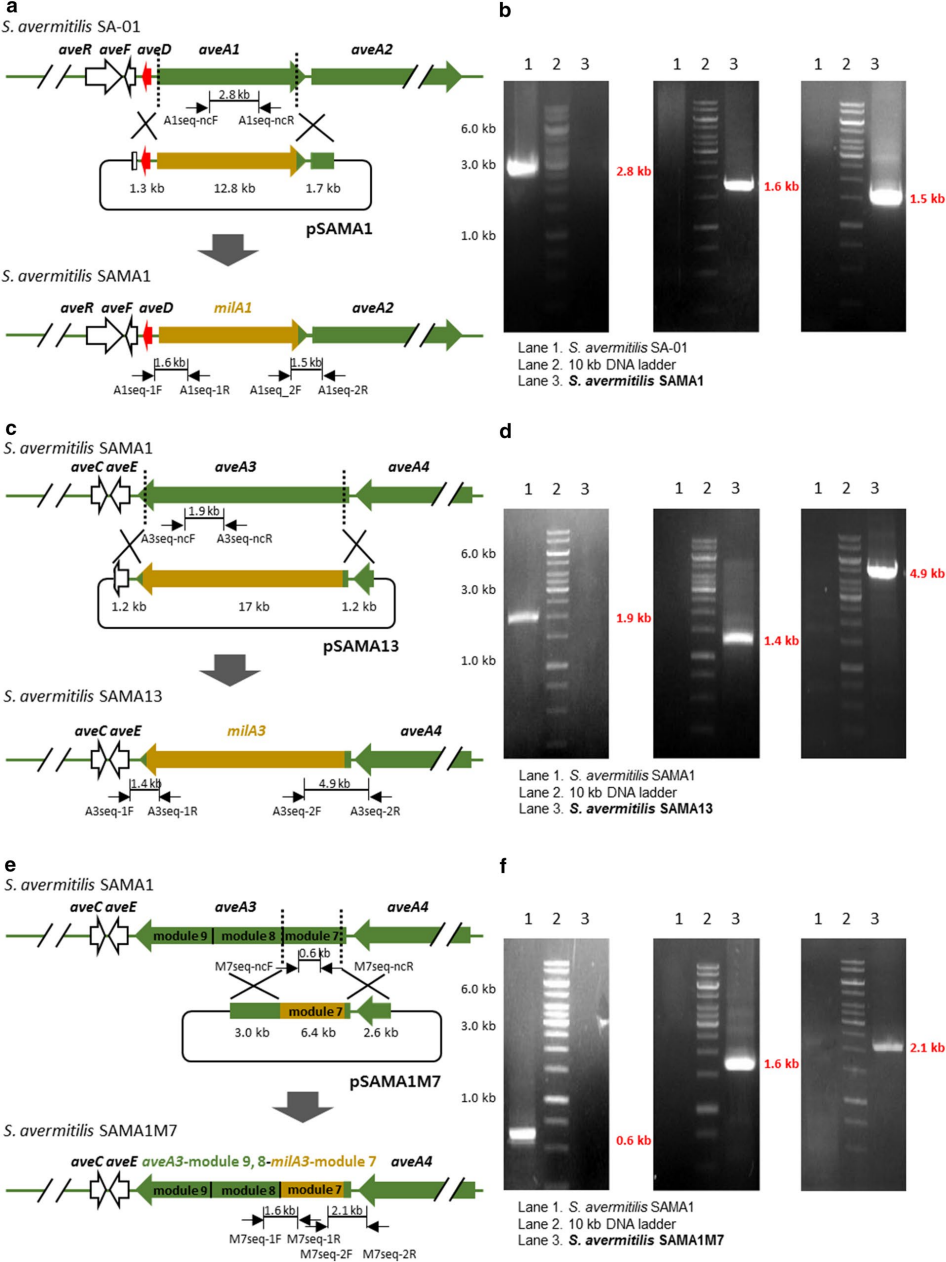
Construction of *S. avermitilis* mutant strains. **a** Schematic diagram of *aveA1* replacement using pSAMA1, and **b** PCR analysis for the confirmation of *S. avermitilis* SAMA1. **c** Schematic diagram of *aveA3* replacement using pSAMA13, and **d** PCR analysis for the confirmation of *S. avermitilis* SAMA13. **e** Schematic diagram of *aveA3*-module7 replacement using pSAMA1M7, and **f** PCR analysis for the confirmation of *S. avermitilis* SAMA1M7

**Fig. 5:**
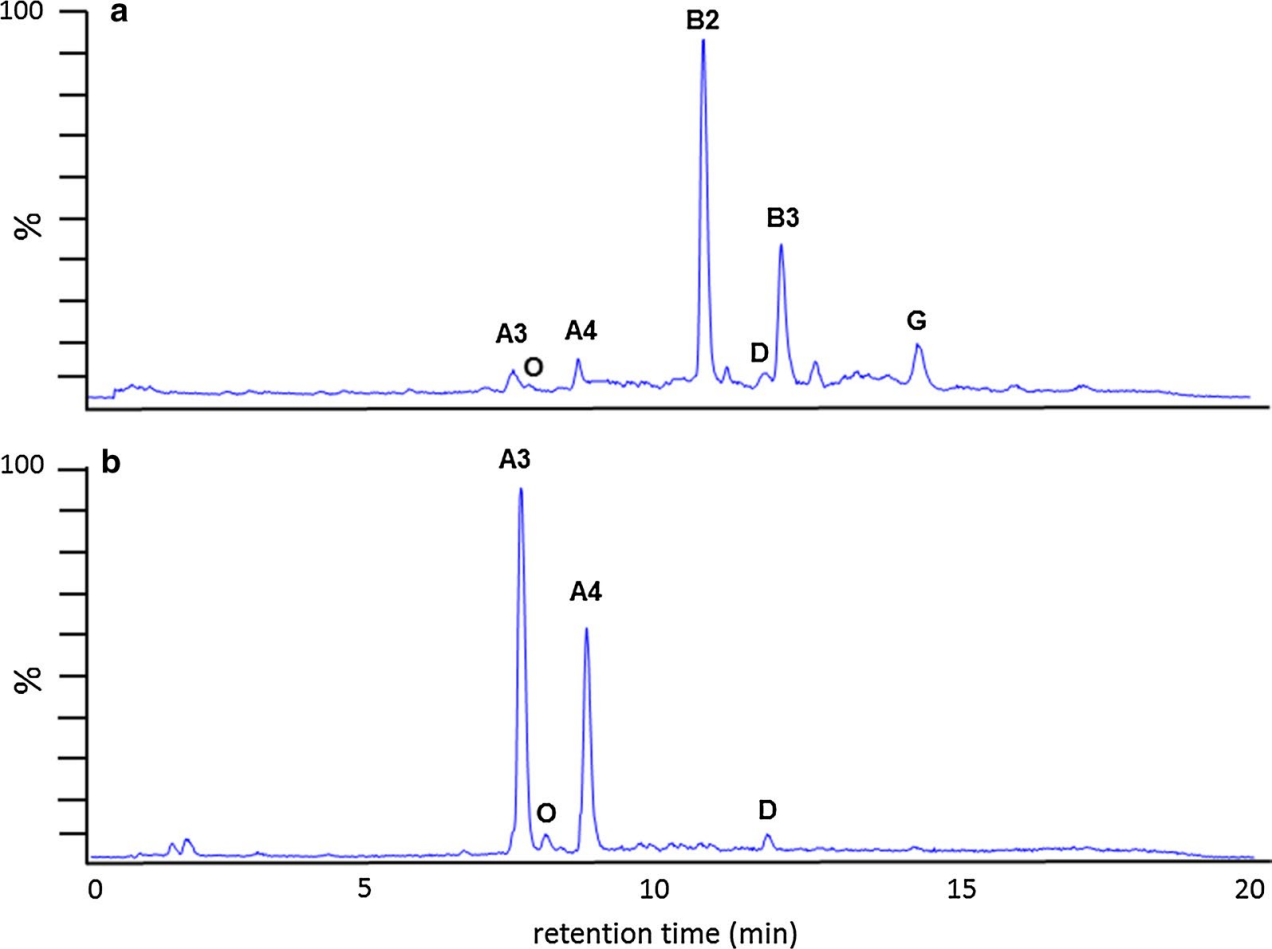
UPLC-qTOF-HR-MS/MS analysis of the extracts obtained from the mutant strains. **a** UPLC-qTOF-MS/MS chromatogram selected for *m/z* = 527.3014 corresponding to milbemycin A3 (A3), *m/z* = 541.3171 corresponding to milbemycin A4 (A4) and milbemycin B2 (B2), *m/z* = 555.3327 corresponding to milbemycin D (D) and milbemycin B3 (B3), *m/z* = 569.3484 corresponding to milbemycin G (G), and *m*/*z* = 789.5158 corresponding to oligomycin A (O), respectively, of culture extracts from the *S. avermitilis* SAMA13 and SAMA1M7 strains. **b** UPLC-qTOF-MS/MS chromatogram selected for *m/z* = 527.3014, 541.3171, 555.3327, and 789.5158 corresponding to milbemycin A3 (A3), milbemycin A4 (A4), milbemycin D (D), and oligomycin A (O), respectively, of culture extracts from the *S. avermitilis* SAMA1M7ΔD strain

To begin, we constructed the mutant strain *S. avermitilis* SAMA1 by replacing *aveA1* in *S. avermitilis* SA-01 with *milA1* from *S. hygroscopicus* subsp. *aureolacrimosus*. The temperature-sensitive *Escherichia coli*-*Streptomyces* shuttle vector pKC1139 [27] containing an apramycin resistance marker was used for gene replacement. The *aveA1* replacement plasmid pSAMA1 was constructed to express the loading module and modules 1 and 2 of MilA1 efficiently by maintaining the promoter region of the *aveA1* gene (Additional file 1: Figure S1a) and to retain the C-terminal docking domain of AveA1 for the maintenance of its interaction with downstream PKS AveA2 (Fig. 3a; Additional file 1: Figure S1a). Plasmid pSAMA1 was transferred by conjugation from *E. coli* ET12567/pUZ8002 [28] to *S. avermitilis* SA-01 and the double crossover mutant was selected by their apramycin-sensitive phenotype which was verified by PCR analysis. The expected 1.6-kb DNA fragment covering the promoter of *aveA1* and the AT domain of the loading module in MilA1 was obtained from the genomic DNA of *S. avermitilis* SAMA1 with the primers A1seq-1F and A1seq-1R (Additional file 2: Table S1), while no PCR product was generated from the genomic DNA of *S. avermitilis* SA-01. Similarly, the 1.5-kb DNA fragment covering the ACP domain of module 2 in MilA1 and the C-terminal docking domain of AveA1 was detected from the genomic DNA of *S. avermitilis* SAMA1 using the primers A1seq-2F and A1seq-2R (Additional file 2: Table S1), whereas no PCR product was observed from the genomic DNA of *S. avermitilis* SA-01. As a negative control, no PCR product corresponding to the internal region of AveA1 was amplified from the genomic DNA of *S. avermitilis* SAMA1 using the primers A1seq-ncF and A1seq-ncR (Additional file 2: Table S1), while a 2.8-kb DNA fragment containing the internal region of AveA1 was detected from the *S. avermitilis* SA-01 genomic DNA (Fig. 4a, b). The sequencing of the PCR products further confirmed that *aveA1* was replaced with *milA1* while the promoter and docking domain regions were maintained as designed. Next, using the replacement plasmid pSAMA13 and a similar DNA replacement approach, the AveA3 in the avermectin PKS of *S. avermitilis* SAMA1 was substituted with MilA3 where the N- and C-terminal docking domains of AveA3 were retained, yielding a mutant strain *S. avermitilis* SAMA13 (Fig. 3a; Additional file 1: Figure S1b). The genotype of the apramycin-sensitive double crossover mutant was confirmed by PCR analysis and sequencing of the PCR products. The predicted 1.4-kb DNA fragment covering the C-terminal docking domain of AveA3 as well as the ACP and part of the KR domains of module 9 in MilA3, and a 4.9-kb DNA fragment covering the part of module 7 (KS, AT, DH, partial ER domains) in MilA3 and the N-terminal docking domain in AveA3 were amplified from the genomic DNA of *S. avermitilis* SAMA13 with primers A3seq-1F/A3seq-1R and A3seq-2F/A3seq-2R, respectively (Additional file 2: Table S1). However, no corresponding PCR products were detected from the genomic DNA of *S. avermitilis* SAMA1. Again, no PCR product containing the internal region of AveA3 was detected from the genomic DNA of *S. avermitilis* SAMA13 using the primers A3seq-ncF and A3seq-ncR (Additional file 2: Table S1), while a 1.9-kb DNA fragment covering the internal region of AveA3 was amplified from the *S. avermitilis* SAMA1 genomic DNA (Fig. 4c, d). The organic extracts obtained from the 14-day-old culture of *S. avermitilis* SAMA13 strain were analyzed by ultra-performance liquid chromatography (UPLC) combined with quadrupole-time of flight high resolution mass spectrometry (qTOF-HR-MS) under negative electrospray ionization (ESI) mode. Peaks corresponding to milbemycins A3 (selected for *m/z* = 527.3014), A4 (selected for *m/z* = 541.3171), B2 (selected for *m/z* = 541.3171), D (selected for *m/z* = 555.3327), B3 (selected for *m/z* = 555.3327), and G (selected for *m/z* = 569.3484) were observed at retention times of 7.5, 8.9, 11.2, 12.8, 13.1, and 14.6 min, respectively (Fig. 5a). The respective retention times and MS/MS fragmentation patterns of milbemycins A3, A4, and D produced by the mutant strain *S. avermitilis* SAMA13 were identical to those of standard milbemycins A3, A4, and D. The identities of other milbemycin congeners obtained were confirmed by their molecular weights and MS/MS fragmentation patterns (see below). The titers of total milbemycins were 291.5 mg/l and the major products were C5-*O*-methylated milbemycins B2, B3, and G (Table 1; Fig. 5a). Only trace amounts of oligomycin A, which is co-produced in *S. avermitilis*, was detected by UPLC-qTOF-HR-MS (Fig. 5a) and also high-performance liquid chromatography (HPLC) analysis (Additional file 3: Figure S2a) under the culture conditions used in this study. The retention time and MS/MS fragmentation pattern of oligomycin A produced by the mutant strain *S. avermitilis* SAMA13 were identical to those of authentic oligomycin A (Additional file 4: Figure S3).

**Table 1:** Production of milbemycins

Strain	Milbemycins (mg/l)						
	A3	A4	D	B2	B3	G	Total
SAMA13	7.3 ± 5.5	1.4 ± 1.1	1.3 ± 0.9	183.7 ± 3.0	76.0 ± 8.9	21.8 ± 5.1	291.5 ± 12.7
SAMA1M7	8.0 ± 0.6	1.4 ± 0.2	1.0 ± 0.1	193.1 ± 43.1	70.5 ± 7.2	17.7 ± 10.1	291.7 ± 58.0
SAMA1M7ΔD (flask)	156.6 ± 15.8	68.0 ± 1.9	22.5 ± 0.4	ND	ND	ND	247.1 ± 14.0
SAMA1M7ΔD (fermenter)	278.9 ± 10.0	98.4 ± 5.9	22.4 ± 9.5	ND	ND	ND	399.7 ± 20.0

*ND* Not detected

We also wished to investigate the effect of replacing module 7 in Ave3 with the corresponding module from MilA3 on the production of the milbemycins. The mutant strain *S. avermitilis* SAMA1M7, in which module 7 of Ave3 was replaced with that of MilA3 while the N-terminal docking domain of Ave3 was retained, was constructed using the plasmid pSAMA1M7 (Fig. 3b; Additional file 1: Figure S1c). The genotype of the *S. avermitilis* SAMA1M7 strain was verified by PCR analysis and sequencing of the PCR products. The 1.6-kb DNA fragment (covering the part of KS domain of module 8 in AveA3 and the part of ACP domain of module 7 in MilA3) and the 2.1-kb DNA fragment (covering the KS domain of module 7 in MilA3 and N-terminal docking domain in AveA3) were detected from the genomic DNA of *S. avermitilis* SAMA1M7 with primers M7seq-1F/M7seq-1R and M7seq-2F/M7seq-2R, respectively (Additional file 2: Table S1). However, no such PCR product was amplified from the genomic DNA of *S. avermitilis* SAMA1. The 0.6-kb DNA fragment corresponding to the part of module 7 in AveA3 was amplified from the genomic DNA of *S. avermitilis* SAMA1 using the primers M7seq-ncF and M7seq-ncR (Additional file 2: Table S1), while no such PCR product was detected from the *S. avermitilis* SAMA1M7 genomic DNA (Fig. 4e, f). The component ratio and amounts of milbemycins produced from *S. avermitilis* SAMA1M7 were similar to those produced from *S. avermitilis* SAMA13 (Table 1).

### Inactivation of *aveD*

**Fig. 6:**
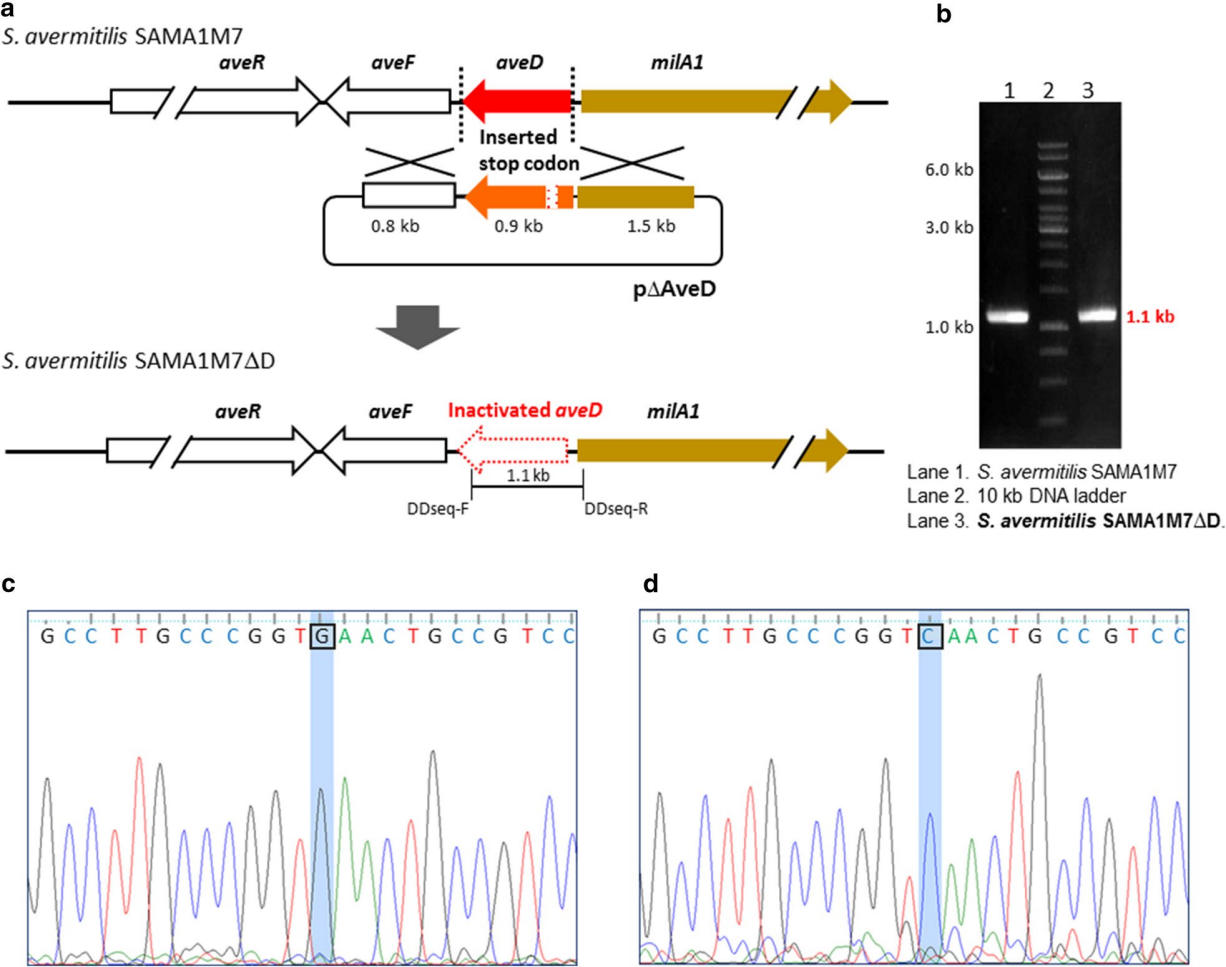
Construction of *S. avermitilis* SAMA1M7ΔD. **a** Schematic diagram for construction of the *aveD* disruption mutant using pΔAveD. **b** PCR analysis for confirmation of the AveD inactivation using genomic DNA from *S. avermitilis* SAMA1M7ΔD. **c** DNA sequences of the *aveD* PCR product of *S. avermitilis* SAMA1M7. **d** DNA sequences of the inactivated *aveD* PCR product of *S. avermitilis* SAMA1M7ΔD. While TCA sequences exist in SAMA1M7 chromosome, the stop codon TGA sequences were generated in SAMA1M7ΔD chromosome

In order to produce milbemycins A3 and A4, the predominant milbemectin components, as major products, the C5-*O*-methyltransferae AveD was inactivated in the *S. avermitilis* SAMA1M7 strain by inserting a stop codon 140-bp downstream from the start codon of *aveD* using the plasmid pΔAveD (Additional file 5: Figure S4). The genotype of the resulting mutant strain *S. avermitilis* SAMA1M7ΔD was confirmed by sequencing the 1.1-kb PCR product covering the engineered stop codon with primers DDseq-F/DDseq-R (Fig. 6; Additional file 2: Table S1). The organic extracts obtained from the *S. avermitilis* SAMA1M7ΔD strain were analyzed by UPLC-qTOF-HR-MS, and peaks corresponding to milbemycins A3, A4, and D were observed (Fig. 5b). The titers of total milbemycins were approximately 247.1 mg/l and the major products were milbemycins A3 and A4 along with small amounts of milbemycin D. As expected, the production of C5-*O*-methylated milbemycins was completely abolished (Table 1; Fig. 5b). Again, only trace amounts of oligomycin A were observed by UPLC-qTOF-HR-MS (Fig. 5b) and HPLC analysis (Additional file 3: Figure S2b).

**Fig. 7:**
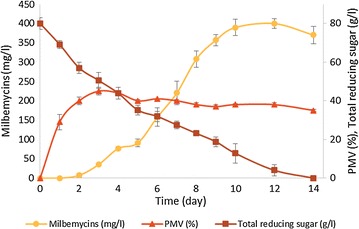
Time course analysis of milbemycin production (), the packed mycelial volume (PMV) value (), and the amount of total reducing sugar () during cultivation of *S. avermitilis* SAMA1M7ΔD in a 5 l fermenter

The production of milbemycins from the *S. avermitilis* SAMA1M7ΔD strain was monitored in the fermenter (5 l) throughout a 14-day incubation period (Fig. 7). The milbemycin production level of the *S. avermitilis* SAMA1M7ΔD strain showed a notable increase between 2 and 10 days in the late exponential and stationary growth phase. It was observed that the carbon sources were continuously consumed during the stationary phase suggesting that carbon sources are required for the milbemycin production and cell maintenance. The milbemycin titers reached 399.7 mg/l after 12 days, which was an approximately 1.6-fold improvement compared to the flask culture. The ratio of milbemycins A3 and A4 was approximately 3:1 along with a small amount of milbemycin D (Table 1).

### Characterization of milbemycins by HR MS/MS analysis

Six milbemycin derivatives show relatively low affinities to protons during positive ESI mode. Although sodium adduct ions tend to be generated with the ion source under positive ESI mode, they were unacceptable for MS/MS detection because of their unstable fragmentation patterns. Therefore, proton loss ions under the negative ESI mode were chosen as the parent ions for MS/MS.

The MS/MS spectra of milbemycins A3, A4, and D obtained from *S. avermitilis* SAMA13, SAMA1M7, and SAMA1M7ΔD were identical to those of authentic milbemycins A3, A4, and D. The MS/MS spectra of milbemycins A3, A4, and D produced from *S. avermitilis* SAMA1M7 and their fragmentation pathways are presented in Additional file 6: Figures S5a, S5b, and S5c, respectively. The molecular formula for milbemycin A3 was confirmed to be C_31_H_44_O_7_ on the basis of the (−)-HRESIMS (high-resolution electrospray ionization mass spectrometry) peak at *m/z* = 527.3034 [M–H]^−^ (calcd 527.3014 for C_31_H_43_O_7_^−^). Milbemycin A3 had its proton loss precursor peak at *m/z* = 527.3034, which was accompanied by several product ions (Additional file 6: Figures S5a). The molecular formula for milbemycin A4 was determined to be C_32_H_46_O_7_ on the basis of (−)-HRESIMS proton loss precursor peak at *m/z* = 541.3170 [M–H]^−^ (calcd 541.3171 for C_32_H_45_O_7_^−^), which was fragmented into several product ions (Additional file 6: Figures S5b). The milbemycin D formula was determined to be C_33_H_48_O_7_ on the basis of (−)-HRESIMS peak at *m/z* = 555.3311 [M–H]^−^ (calcd 555.3327 for C_33_H_47_O_7_^−^), which also produced several product ions (Additional file 6: Figures S5c). There are fragment ions including *m/z* = 321.1860 and 109.0295 common to all three compounds, and the existence of characteristic fragmentation patterns of milbemycins A3, A4, and D (*m/z* = 143.1078, 157.1234, and 171.1391, respectively) shows that the structural difference stems from the methyl, ethyl, and isopropyl moieties on C25. In addition, the corresponding fragment ions within the three milbemycins A3, A4, and D show a difference of 14 Da between each other, and these could be used as diagnostic ions for the structural determination of milbemycins A3, A4, and D, respectively. The fragmentation of milbemycins A3, A4, and D shows similar patterns resulting from the repeated loss of water, carbon dioxide, hydrogen gas, and ethylene (Additional file 6: Figures S5a, S5b, and S5c).

The MS/MS spectra of milbemycins B2, B3, and G obtained from *S. avermitilis* SAMA1M7 and their fragmentation pathways are presented in Additional file 6: Figures S5d, S5e, and S5f, respectively. The spectra obtained from *S. avermitilis* SAMA13 were identical to those from *S. avermitilis* SAMA1M7. The identities of milbemycins B2, B3, and G were confirmed by their molecular weights and the comparison of their MS/MS fragmentation patterns with those of authentic milbemycins A3, A4, and D. The molecular formula for milbemycin B2 was determined to be C_32_H_46_O_7_ on the basis of the (−)-HRESIMS proton loss precursor peak at *m/z* = 541.3168 [M–H]^−^ (calcd 541.3171 for C_32_H_45_O_7_^−^). This peak was fragmented into several product ions (Additional file 6: Figures S5d). The milbemycin B3 formula was established as C_33_H_48_O_7_ on the basis of the (−)-HRESIMS peak at *m/z* 555.3312 [M–H]^−^ (calcd 555.3327 for C_33_H_47_O_7_^−^), which was accompanied by several product ions (Additional file 6: Figures S5e). The milbemycin G formula was shown to be C_34_H_50_O_7_ on the basis of the (−)-HRESIMS peak at *m/z* = 569.3440 [M–H]^−^ (calcd 569.3484 for C_34_H_49_O_7_^−^), which also produced several product ions (Additional file 6: Figures S5f). Again, the common fragment ions (*m/z* = 321.1860 and 109.0295) were detected, and the existence of the characteristic fragmentation patterns of milbemycins B2, B3, and G (*m/z* = 143.1078, 157.1234, and 171.1391, respectively) indicates that their structural difference occurred due to the C25 moieties. Similarly, the difference of 14 Da in the corresponding fragment ions within three milbemycins B2, B3, and G could be used as diagnostic indicators for milbemycins B2, B3, and G, respectively. Unlike the fragmentation patterns of milbemycins A3, A4, and D, loss of CH_3_OH as a neutral molecule from the C5-methoxy group occurred in these C5-*O*-methylated congeners. These detailed results of qTOF-HR-MS/MS analysis unequivocally confirmed the identities of milbemycins produced from the *S. avermitilis* mutant strains.

## Discussion

Interestingly, the nucleotide sequences of *milA1* cloned from *S. hygroscopicus* subsp. *aureolacrimosus* are identical to those reported from *S. bingchenggensis* [15], and only 68 nucleotide sequences of *milA3* (~17.5-kb) from *S. hygroscopicus* subsp. *aureolacrimosus* are changed compared to the *S. bingchenggensis* sequences. Therefore, it seems that the horizontal transfer of PKS genes occurred between these two *Streptomyces* strains although we did not sequence other milbemycin biosynthetic genes or the genes located between *milA3* and *milA1*. The sequences of *milA3* of *S. hygroscopicus* subsp. *aureolacrimosus* have been deposited in the GenBank database under the accession number KY056568.

Although it has been demonstrated over the last two decades that the engineered PKSs can produce the desired compounds, low production of novel natural products is often encountered mainly due to the disruption of the overall enzyme integrity/interaction and inefficient substrate recognition/channeling along the engineered PKS machinery [20]. However, the well-designed assembly of structurally and functionally related PKSs, such as avermectin and milbemycin PKS, in a previously optimized high-producing strain can produce large quantities of desired products as demonstrated in this study. The host strain used in this study, *S. avermitilis* SA-01, produces approximately 1.1 mM of the avermectins which corresponds to approximately 470 mg/l of the milbemycins on a molar basis in the fermenter culture. Although the hybrid PKS constructed in this study did not fully reproduce the maximum production capacity of the host strain, the engineered strain *S. avermitilis* SAMA1M7ΔD produced approximately 247 and 400 mg/l of milbemycins A3, A4, and D in the flask and fermenter culture, respectively. The productivity of the hybrid PKS varies mainly depending on the choice of promoter or host strain, fusion strategies (e.g. fusion sites for the domain/module substitution and the use of proper docking domains), and sometimes the similarity of mixed PKSs. The similar high titers of milbemycins between *S. avermitilis* SAMA13 and SAMA1M7 strains imply that the similarity between milbemycin and avermectin PKSs allowed us to construct functional and productive hybrid PKSs either on modular or subunit levels with maintenance of only the docking domains, although It has been suggested that not only the docking domain but also other domains such as KS and AT domains participate in molecular recognition between PKSs [29].

Similarly, when a midecamycin biosynthetic gene was introduced into a tylosin-overproducing *Streptomyces fradiae* from which its native tylosin biosynthetic gene cluster was deleted, a substantial level of midecamycin analog (1 g/l) was obtained [30]. In another example, the heterologous expression of the tetracenomycin biosynthetic genes in an industrial monensin producer *Streptomyces cinnamonensis* led to the high titer production of tetracenomycins [31]. These previous results [22, 23, 30, 31] together with ours shows that the overproducing feature of the industrial strains is mainly caused by the mutations in non-biosynthetic genes and can be utilized with heterologous or engineered PKSs. Although the titers of milbemycins A3 and A4 in the mutant strain of *S. bingchenggensis* were reported to reach 1450 mg/l [18], it is notable that *S. avermitilis* is one of most well-studied *Streptomyces* species [32] and the feasibility of the *S. avermitilis* strain in which active biosynthetic gene clusters were deleted for the production of a range of natural products has been successfully demonstrated [33, 34]. Therefore, although the results presented here were obtained using a moderately improved *S. avermitilis* mutant, application of the same strategy to higher producing industrial strains and further optimization by metabolic engineering and synthetic biology strategies may allow for more improved production of the milbemycins. Most importantly, the well-established genetic tools in *S. avermitilis* will facilitate further exploration of novel milbemycin analogs with improved properties.

It is known that AveC catalyzes the spirocyclization of a C17, C25-dihydroxy C21-ketone polyketide intermediate containing a C23-hydroxy group to generate avermectin “2” series and the optional dehydration to yield avermectin “1” series with double bond between C22–C23 in *S. avermitilis* [17]. The hybrid PKS, where AveA1 was replaced with MilA1 which contains fully active DH-ER-KR domains in module 2, biosynthesizes a polyketide intermediate without the C23-hydroxy group (Fig. 3). Our results along with previous studies where the loading module and module 2 of AveA1 or the entire AveA1 were replaced with the respective milbemycin PKS domains or subunit [22, 23] showed that AveC is substrate-flexible and can successfully catalyze the spirocyclization of this modified intermediate producing the desired compounds.

Previous studies showed that the replaced milbemycin loading module from *S. bingchenggensis* accepts only acetyl-CoA and propionyl-CoA to generate 25-methyl and 25-ethyl avermectin analogs in *S. avermitilis* [22, 23]. However, we observed the production of small amounts of milbemycins D and G which contain an isopropyl side chain at C25 (Fig. 5) implying that the loading module of milbemycin-avermectin hybrid PKS shows certain degree of substrate flexibility in the heterologous host as in the native producing strain. Because the sequences of MilA1 from *S. hygroscopicus* subsp. *aureolacrimosus* are identical to those of *S. bingchenggensis*, this observed discrepancy was probably simply due to the detection method used. While the produced metabolites were monitored only by HPLC in the previous studies [22, 23], a highly sensitive UPLC-qTOF-HR-MS analysis was employed in this study. The commercial milbemectin is a mixture of 70% milbemycin A4 and 30% milbemycin A3. However, *S. avermitilis* SAMA1M7ΔD produced more milbemycin A3 than A4 (Table 1). Therefore, further engineering of the AT domain in the milbemycin loading module to alter its substrate preference from acetyl-CoA to propionyl-CoA is required to meet the ratio of milbemycin A3/A4 in commercial milbemectin. To this end, the following approaches can be applied: replacement of the AT domain with one that prefers propionyl-CoA, site-specific mutagenesis based on molecular docking simulation, or direct evolution to reprogram AT domain specificity [20].

## Conclusions

These results demonstrate that large quantities of milbemycins can be produced in the avermectin high-producing *S. avermitilis* by expression of the engineered PKS which was designed to minimize adverse effects on protein integrity and interactions. Application of the same strategy in the industrial *S. avermitilis* will further increase the milbemycin titer and also allow for generating novel milbemycin analogs with improved properties in sufficient amounts for further development.

## Methods

### Bacterial strains, plasmids, culture medium, and reagents

All bacterial strains and plasmids used in this study are listed in Table 2. *E. coli* DH5α was used as a subcloning host for performing plasmid constructions and cultured at 37 °C in Luria–Bertani medium [35] supplemented with suitable antibiotics. *S. avermitilis* SA-01, an avermectin high-producing strain, was employed as an initial strain for the construction of the milbemycin-producing strain. Constructs for gene replacement were generated with pKC1139 [27]. Non-methylating *E. coli* ET12567/pUZ8002 was used as a donor strain in conjugal transfer, and the conjugal transfer of recombinant plasmids between *Streptomyces* and *E. coli* was conducted on solid Mannitol-Soy flour (MS) medium [28]. Inorganic Salt Starch (ISP4) agar medium [36] was utilized for sporulation and generation of *S. avermitilis* and *S. hygroscopicus* subsp. *aureolacrimosus*. Isolation of genomic DNA from *S. avermitilis* and *S. hygroscopicus* subsp. *aureolacrimosus* were performed according to standard protocol using the culture grown in ISP2 liquid medium [28]. A genomic DNA from *S. hygroscopicus* subsp. *aureolacrimosus* NRRL 5739, a milbemycin-producing strain, was used as a template for the *milA1* and *milA3* genes. Since the nucleotide sequences of the milbemycin biosynthetic genes of the *S. hygroscopicus* subsp. *aureolacrimosus* were not known, the deoxyoligonucleotide primers for the cloning of *milA1* and *milA3* were designed based on the sequences of *S. bingchenggensis* BCW-1 (GenBank: CP002047.1). The DNA fragments used for the construction of replacement or inactivation plasmids were amplified by PCR with the corresponding primers (Additional file 2: Table S1) and template DNAs. PCR was performed by using GXL DNA polymerase (Takara, Shiga, Japan) acceding to the manufacturer’s recommended condition. All PCR products were cloned into plasmid Litmus 28 (New England Biolabs, Ipswich, USA), pGem-T Easy (Promega, Madison, USA), or pCR2.1 TOPO (Invitrogen, Carlsbad, USA) and sequenced to confirm their authenticity. DNA sequencing was performed by Cosmogenetech (Seoul, Korea). The authentic milbemycins A3, A4, and D and oligomycin A were purchased from BioAustralis Fine Chemicals (Sydney, Australia).

**Table 2:** Strains and plasmids used in this study

Strain/plasmid	Description	Source/References
*Strain*		
*E. coli* DH5α	Host for plasmids construction and subcloning	New England Biolabs
*E. coli* ET12567/pUZ8002	Non-methylating ET12567 containing non-transmissible RP4 derivative plasmid pUZ8002	Kieser et al. [28]
*S. hygroscopicus* subsp. *aureolacrimosus* NRRL 5739	The milbemycin-producing strain	Takiguchi et al. [6]
*S. avermitilis* SA-01	Industrial avermectin-producing *S. avermitilis*	This study
*S. avermitilis* SAMA1	Mutant strain of *S. avermitilis* SA-01 with AveA1 replaced by MilA1	This study
*S. avermitilis* SAMA13	Mutant strain of SAMA1 with AveA3 replaced by MilA3	This study
*S. avermitilis* SAMA1M7	Mutant strain of SAMA1 with AveA3-module7 replaced by MilA3-module7	This study
*S. avermitilis* SAMA1M7ΔD	AveD disruption mutant of SAMA1M7	This study
*Plasmid*		
pLitmus28	*E*. *coli* vector for subcloning, Amp^R^	New England Biolabs
pGEM T easy	*E*. *coli* vector for cloning PCR products, Amp^R^	Promega
pCR2.1 TOPO	*E*. *coli* vector for cloning PCR products, Amp^R^	Invitrogen
pKC1139	Temperature-sensitive *E*. *coli*-*Streptomyces* shuttle vector containing *oriT* for gene substitution and disruption, Apr^R^	Bierman et al. [27]
pSAMA1	pKC1139-based plasmid containing *aveA1* upstream fragment, entire *milA1* fragment, and *aveA1* downstream fragment	This study
pSAMA13	pKC1139-based plasmid containing *aveA3* upstream fragment, entire *milA3* fragment, and *aveA3* downstream fragment	This study
pSAMA1M7	pKC1139-based plasmid containing *aveA3*-module 7 upstream fragment, *milA3*-module 7 fragment, and *aveA3*-module 7 downstream fragment	This study
pΔAveD	pKC1139-based plasmid containing inactivated *aveD* fragment	This study

### Construction of AveA1/AveA3 replacement mutant strain *S. avermitilis* SAMA13

For the replacement of the *aveA1* gene with *milA1* in *S. avermitilis* SA-01, a plasmid pSAMA1 was constructed. The DNA segments encoding MilA1 (Genbank: CP002047.1, 10777186-10790000 nt) were obtained by PCR amplification. The 8.6-kb fragment (Genbank: CP002047.1, 10777186-10785834 nt) and the 3.8-kb fragment (Genbank: CP002047.1, 10785829-10789650 nt) were amplified with primers A1-1F/A1-1R and A1-2F/A1-2R, respectively. On the other hand, a 1.0-kb fragment including MilA1-ACP2 (Genbank: CP002047.1, 10789645-10790000 nt) and the C-terminal docking domain of AveA1 was artificially synthesized. Additionally, a 1.3-kb upstream flanking region of *aveA1* containing the promoter region of *aveA1* as well as a 1.1-kb downstream flanking region of *aveA1* containing C-terminal docking domain of AveA1 were amplified using primer pairs A1LF/A1LR and A1RF/A1RR, respectively. The stepwise assembly of the PCR products and the synthesized DNA fragment was carried out by conventional cloning procedures or the infusion ligation method, which relies on intramolecular recombination between the adjacent homologous DNA sequences in pCR2.1 TOPO according to the manufacturer’s protocol (Clontech Laboratories Inc, Mountain View, USA). The final recombinant plasmid pSAMA1 was constructed by transfer of the *Xba*I/*Hind*III fragment from pCR2.1 TOPO into pKC1139. After non-methylating *E. coli* ET12567/pUZ8002 was transformed with the pSAMA1, conjugal transfer of the recombinant plasmid from the *E. coli* into *S. avermitilis* SA-01 was performed as described previously [28]. Exconjugants selected based on apramycin resistance were verified by PCR analysis for single crossover homologous recombination and propagated in antibiotic-free ISP4 medium for second round of homologous recombination. The desired *milA1* substitution mutant which showed no resistance to apramycin was confirmed by sequencing of the PCR product obtained using the mutant genomic DNA as a template, generating *S. avermitilis* SAMA1.

Plasmid pSAMA13 was constructed for the replacement of the *aveA3* gene with *milA3* in *S. avermitilis* SAMA1. The DNA fragment containing *milA3* (Genbank: CP002047.1, 10855562-10872660 nt) was prepared by PCR in three fragments using the listed primer pairs (A3-1F/A3-1R, A3-2F/A3-2R, A3-3F/A3-3R; Additional file 2: Table S1) and ligated into pGem-T Easy, respectively, to provide a 3.7-kb (Genbank: CP002047.1, 10868959-10872660 nt), a 6.7-kb (Genbank: CP002047.1, 10862242-10868964 nt), and a 6.7-kb (Genbank: CP002047.1, 10855562-10862247 nt) fragment. Meanwhile, the primer pairs, A3LF/A3LR and A3RF/A3RR were designed to amplify *aveA3* flanking regions which contain the C- and N-terminal docking domain sequences of AveA3 from the *S. avermitilis* SA-01, resulting in an *EcoR*I/*Mfe*I fragment (1.2-kb) carrying the downstream region of *aveA3* and a *Mfe*I/*Hind*III fragment (1.2-kb) carrying the upstream region of *aveA3*, respectively. For seamless ligation of *aveA3* flanking regions to both ends of the *milA3* gene, nucleotide sequences were changed to create *Mfe*I sites in which the amino acid sequences were not altered (CCATCG and CAGCTC → CAATTG; Additional file 1: Figure S1b). The fragment containing the C-terminal docking domain of AveA3, the fragments encoding MilA3, and the fragment containing N-terminal docking domain of AveA3 were combined into Litmus28 through a series of subcloning, and the 19-kb *Eco*RI/*Hind*III fragment carrying the whole combined DNA was transferred into the same sites of pKC1139 to generate pSAMA13. Following the procedure described above [28], pSAMA13 was transformed into the strain *S. avermitilis* SAMA1, and the double crossover mutants were selected. After verification by PCR analysis, the obtained *milA3* substitution mutant was designated as *S. avermitilis* SAMA13.

### Construction of AveA1/module7 replacement mutant strain *S. avermitilis* SAMA1M7

In order to swap the *aveA3*-module7 for the *milA3*-module7 in *S. avermitilis* SAMA1, plasmid pSAMA1M7 was constructed. PCR was performed to amplify a 3.0-kb *Xba*I/*Spe*I fragment containing the downstream region of *aveA3*-module7 and a 2.6-kb *Spe*I/*Hind*III fragment containing the upstream region and N-terminal docking domain sequences of *aveA3* using primer pairs M7LF/M7LR and M7RF/M7RR, respectively. A primer pair M7-F/M7-R was utilized for amplification of a 6.5-kb fragment carrying *milA3*-module7 region (Genbank: CP002047.1, 10855552-10862085 nt) from *S. hygroscopicus* subsp. *aureolacrimosus* genomic DNA. After the upstream fragment of *aveA3*-module7, a *milA3*-module7 fragment, and downstream fragment of *aveA3*-module7 were inserted to pCR2.1 TOPO, the entire combined fragment was transferred to pKC1139 using *Xba*I/*Hind*III, resulting in pSAMA1M7. The plasmid pSAMA1M7 was introduced into the chromosome of strain *S. avermitilis* SAMA1 following the procedures described [28]. The resulting double crossover mutant, namely *S. avermitilis* SAMA1M7, were verified by PCR amplification and sequencing of the PCR products.

### Construction of AveD inactivation mutant strain *S. avermitilis* SAMA1M7ΔD

Plasmid pΔAveD carrying the truncated *aveD* gene was prepared for the inactivation of AveD. A 1.6-kb fragment carrying the partial upstream region of *aveD* was amplified by PCR using primers DD-1F/DD-1R from *S. avermitilis* SAMA1M7 genomic DNA, in which stop codon TGA is generated through a nucleotide substitution 140-bp downstream from the start codon of *aveD* (Additional file 5: Figure S4). Another 1.5-kb fragment which contains the partial downstream region of *aveD* was obtained by PCR amplification with primers DD-2F/DD-2R, possessing 20-bp overlapping region with the 1.6-kb fragment. Since the fragments additionally contain homologous sequences with pCR2.1 TOPO, the fragments were ligated into pCR2.1 TOPO with the infusion ligation method. The *Xba*I/*Hind*III fragment was transferred into the same sites of pKC1139 to construct pΔAveD. The AveD disruption in *S. avermitilis* SAMA1M7 via double crossover homologous recombination was achieved according to a previously described protocol [28] to give *S. avermitilis* SAMA1M7ΔD. To genetically verify the disruption, PCR reaction was performed using genomic DNA of the mutant, and the sequencing data of the PCR products were compared to the sequence of the original *aveD* gene.

### Production of milbemycins

*Streptomyces avermitilis* mutant strains were precultivated in seed medium (soluble starch 30 g/l, yeast extract 15 g/l, corn steep liquor 5 g/l, and KH_2_PO_4_ 0.4 g/l, pH 7.2) at 28 °C for 40–48 h using a rotary shaker at 200 rpm. After a portion of the preculture (5% v/v) was transferred into a 250 ml Erlenmeyer flask containing 25 ml main medium (soluble starch 80 g/l, soybean flour 10 g/l, skim milk 15 g/l, and KH_2_PO_4_ 0.5 g/l, KCl 10 g/l, pH 7.0), cultivation was carried out for the production of milbemycins at 28 °C for 14 days at 230 rpm. The resulting culture broth was mixed with an equal volume of acetonitrile:methanol = 1:1 solution or ethyl acetate, and sonication was also conducted in an ultrasonic cleaner for 10 min. The resultant mixture was centrifuged for 5 min and the supernatant was analyzed. For the production of milbemycins in a fermenter, a seed medium composed of glucose monohydrate 20 g/l, soybean flour 15 g/l, yeast extract 5 g/l, and corn steep powder 3 g/l with pH 7.0 was used. Respective strains were cultivated in two 1 l Erlenmeyer flasks containing 100 ml seed medium at 28 °C using a shaking incubator at 200 rpm. After cultivation for 24 h, 150 ml of seed cultures was transferred into 5 l fermenter (Biotron GX, Hanil science medical, Daejeon, Korea) containing 3 l main medium. The culture was grown at 28 °C and dissolved oxygen level was maintained over 30% through aeration in 0.5–1 vvm and varying stirring speed. The pH was maintained at 6.8 during idiophase. Cell growth was estimated by measuring the packed mycelial volume (PMV) from 10 ml fermented broth centrifuged in a 15 ml conic scale tube at 3000 rpm for 10 min. After acid hydrolysis of the supernatant of the fermented broth, total reducing sugar was measured by dinitrosalicylic acid method [37]. The resulting culture broth was extracted as described above and analyzed.

### HPLC and UPLC-qTOF-HR-MS/MS analysis of milbemycins

The organic extracts were applied to HPLC coupled with a photodiode array (PDA) detector and UPLC combined with a quadrupole-time of flight high resolution mass spectrometry (qTOF-HR-MS). HPLC was performed with analytical Phenomenex Luna (4.6 × 100 mm, 5 μm) column on an YL9100 HPLC system (YL Instrument Co, Anyang, Korea) consisting of a YL9110 gradient pump coupled with a YL9160 PDA detector with 1024 channels. A gradient elution using solvent A (0.05% trifluoroacetic acid in water) and solvent B (acetonitrile) as the mobile phase at a flow rate of 1 ml/min was applied. The gradient conditions were as follows: 0–5 min, linear at 40% B; 5–35 min, linear from 40 to 90% B; 35–55 min held at 90% B, then returned to 40% B in 5 min. The injection volume was 20 μl. The absorption spectra of eluted compounds were scanned within 190–600 nm using the in-line PDA detector monitored at 245 nm for milbemycin A3, A4, D, B2, B3, and G. Authentic milbemycins A3, A4, and D (BioAustralis Fine Chemicals), were used to generate calibration curves. The reported production levels of milbemycins are the average based on five separate cultivations and extractions.

The six milbemycins were further identified with a Waters XEVO^®^ G2S Q-TOF mass spectrometer coupled with a Waters Acquity UPLC^®^ system equipped with a Xselect^®^ CSH column XP (2.1 × 100 mm, 2.5 μm) consisting of an Acquity I-Class system. A gradient elution using solvent A (water) and solvent B (acetonitrile) as the mobile phase at a flow rate of 0.4 ml/min was applied. The gradient conditions were as follows: 0–1 min, linear at 50% B; 1–7 min, linear from 50 to 75% B; 7–15 min, linear from 75 to 90% B; 15–18 min held at 90% B, then returned to 50% B in 2 min. The injection volume was 4 μl. The MS system was operated in electrospray ionization (ESI) with a negative ionization mode. The typical operating parameters were as follows: analyser, resolution mode; capillary voltage (volt.), 2.5 kV; sampling cone volt., 25 V; source temperature (temp.), 120 °C, source offset temp., 80 °C; desolvation temp., 300 °C; cone gas flow, 10 l/h; desolvation gas flow, 600 l/h. The analyzer was operated with an extended dynamic range at 10 000 resolution (fwhm at *m/z* 554) with an acquisition time of 0.1 s. Leucine encephalin was used as a lockspray and a reference material for mass spectrometer tuning and calibration. Leucine enkephalin (400 pg/µl, acetonitrile:water (0.1% formic acid), 50:50 by volume con) was infused at a rate of 5 µl/min for mass correction. Mass spectra were acquired with a scan range of 50–700 amu with scan time 0.1 s. For MS/MS analysis, helium collision gas was introduced in accordance with the manufacturer’s recommendations. The MS/MS fragment spectra were produced using collision energy 20 eV for set mass values *m/z* = 527.3014, 541.3171, 555.3327, and 569.3484. MassLynx V4.1 software (Waters) was used for instrument control, acquisition, and sample analysis (Waters Co, Milford, USA).

## Additional files

**Additional file 1**: **Figure S1**. The engineered fusion sites for hybrid PKSs.

**Additional file 2**: **Table S1**. Primers used for construction of plasmids and verification of mutant strains.

**Additional file 3**: **Figure S2**. HPLC analysis of milbemycins produced from *S. avermitilis* mutant strains and authentic standard milbemycins.

**Additional file 4:** **Figure S3** MS/MS spectrum of oligomycin A produced from *S. avermitilis* mutant strains.

**Additional file 5**:** Figure S4**. Sequence of inactivated *aveD*.

**Additional file 6**: **Figure S5**. MS/MS spectra of milbemycins produced from *S. avermitilis* mutant strains.

## Abbreviations

ACP: acyl carrier protein
AT: acyltransferase
DH: dehydratase
ER: enoylreductase
KR: ketoreductase
KS: ketosynthase
PKS: polyketide synthase
PMV: packed mycelial volume
ESI: electrospray ionization
HPLC: high-performance liquid chromatography
HRESIMS: high-resolution electrospray ionization mass spectrometry
PDA: photodiode array
qTOF-HR-MS: quadrupole-time of flight high resolution mass spectrometry
UPLC: ultra-performance liquid chromatography

## References

[1] Burg RW, Miller BM, Baker EE, Birnbaum J, Currie SA, Hartman R, Kong YL, Monaghan RL, Olson G, Putter I, Tunac JB, Warrick H, Stapley EO, Oiwa R, Omura S (1979). Avermectins, new family of potent anthelmintic agents: producing organism and fermentation. Antimicrob Agents Chemother.

[2] Egerton JR, Ostlind DA, Blair LS, Eary CH, Suhayda D, Cifelli S, Riek RF, Campbell WC (1979). Avermectins, new family of potent anthelmintic agents: efficacy of the B1a component. Antimicrob Agents Chemother.

[3] Ikeda H, Omura S (1997). Avermectin biosynthesis. Chem Rev.

[4] Shoop W, Soll M, Vercruysse J, Rew RS (2002). Ivermectin, abamectin and eprinomectin. Macrocyclic lactones in antiparasitic therapy.

[5] Thuan NH, Pandey RP, Sohng JK (2014). Recent advances in biochemistry and biotechnological synthesis of avermectins and their derivatives. Appl Microbiol Biotechnol.

[6] Takiguchi Y, Mishima H, Okuda M, Terao M, Aoki A, Fukuda R (1980). Milbemycins, a new family of macrolide antibiotics: fermentation, isolation and physico-chemical properties. J Antibiot.

[7] Xiang WS, Wang JD, Wang XJ, Zhang J (2007). Two new β-class milbemycins from *Streptomyces bingchenggensis*: fermentation, isolation, structure elucidation and biological properties. J Antibiot.

[8] Xiang WS, Wang JD, Fan HM, Wang XJ, Zhang J (2008). New seco-milbemycins from *Streptomyces bingchenggensis*: fermentation, isolation and structure elucidation. J Antibiot.

[9] Shoop WL, Mrozik H, Fisher MH (1995). Structure and activity of avermectins and milbemycins in animal health. Vet Parasitol.

[10] Lumaret JP, Errouissi F, Floate K, Rombke J, Wardhaugh K (2012). A review on the toxicity and non-target effects of macrocyclic lactones in terrestrial and aquatic environments. Curr Pharm Biotechnol.

[11] Putter I. Milbemycin compounds as anthelmintic agents. Patent US 4144352 A; 1979.

[12] Takeshiba H, Sato K, Yanai T, Yokoi S, Ichinose R. Insecticidal milbemycin derivative having oxime group-containing substituent group at 13-position. Patent JP 09143183; 1997.

[13] Jung M, Saito A, Buescher G, Maurer M, Graf JF, Vercruysse J, Rew RS (2002). Milbemycin oxime. Macrocyclic lactones in antiparasitic therapy.

[14] He Y, Sun Y, Liu T, Zhou X, Bai L, Deng Z (2010). Cloning of separate meilingmycin biosynthesis gene clusters by use of acyltransferase-ketoreductase didomain PCR amplification. Appl Environ Microbiol.

[15] Wang XJ, Yan YJ, Zhang B, An J, Wang JJ, Tian J, Jiang L, Chen YH, Huang SX, Yin M, Zhang J, Gao AL, Liu CX, Zhu ZX, Xiang WS (2010). Genome sequence of the milbemycin-producing bacterium *Streptomyces bingchenggensis*. J Bacteriol.

[16] Ikeda H, Nonomiya T, Usami M, Ohta T, Omura S (1999). Organization of the biosynthetic gene cluster for the polyketide anthelmintic macrolide avermectin in *Streptomyces avermitilis*. Proc Natl Acad Sci USA.

[17] Sun P, Zhao Q, Yu F, Zhang H, Wu Z, Wang Y, Wang Y, Zhang Q, Liu W (2013). Spiroketal formation and modification in avermectin biosynthesis involves a dual activity of AveC. J Am Chem Soc.

[18] Wang XJ, Wang XC, Xiang WS (2009). Improvement of milbemycin-producing *Streptomyces bingchenggensis* by rational screening of ultraviolet-and chemically induced mutants. World J Microbiol Biotechnol.

[19] Zhang J, An J, Wang JJ, Yan YJ, He HR, Wang XJ, Xiang WS (2013). Genetic engineering of *Streptomyces bingchenggensis* to produce milbemycins A3/A4 as main components and eliminate the biosynthesis of nanchangmycin. Appl Microbiol Biotechnol.

[20] Kim E, Moore BS, Yoon YJ (2015). Reinvigorating natural product combinatorial biosynthesis with synthetic biology. Nat Chem Biol.

[21] Rodriguez E, Hu Z, Ou S, Volchegursky Y, Hutchinson CR, McDaniel R (2003). Rapid engineering of polyketide overproduction by gene transfer to industrially optimized strains. J Ind Microbiol Biotechnol.

[22] Huang J, Chen AL, Zhang H, Yu Z, Li MH, Li N, Lin JT, Bai H, Wang JD, Zheng YG (2015). Gene replacement for the generation of designed novel avermectin derivatives with enhanced acaricidal and nematicidal activities. Appl Environ Microbiol.

[23] Zhang J, Yan YJ, An J, Huang SX, Wang XJ, Xiang WS (2015). Designed biosynthesis of 25-methyl and 25-ethyl ivermectin with enhanced insecticidal activity by domain swap of avermectin polyketide synthase. Microb Cell Fact.

[24] Ikeda H, Wang LR, Ohta T, Inokoshi J, Omura S (1998). Cloning of the gene encoding avermectin B 5-*O*-methyltransferase in avermectin-producing *Streptomyces avermitilis*. Gene.

[25] Gokhale RS, Tsuji SY, Cane DE, Khosla C (1999). Dissecting and exploiting intermodular communication in polyketide synthases. Science.

[26] Broadhurst RW, Nietlispach D, Wheatcroft MP, Leadlay PF, Weissman KJ (2003). The structure of docking domains in modular polyketide synthases. Chem Biol.

[27] Bierman M, Logan R, O’Brien K, Seno ET, Rao RN, Schoner BE (1992). Plasmid cloning vectors for the conjugal transfer of DNA from *Escherichia coli* to *Streptomyces* spp.. Gene.

[28] Kieser T, Bibb MJ, Buttner MJ, Chater KF, Hopwood DA (2000). Practical *Streptomyces* genetics.

[29] Kapur S, Chen AY, Cane DE, Khosla C (2010). Molecular recognition between ketosynthase and acyl carrier protein domains of the 6-deoxyerythronolide B synthase. Proc Natl Acad Sci USA.

[30] Rodriguez E, Ward S, Fu H, Revill WP, McDaniel R, Katz L (2004). Engineered biosynthesis of 16-membered macrolides that require methoxymalonyl-ACP precursors in *Streptomyces fradiae*. Appl Microbiol Biotechnol.

[31] Li C, Hazzard C, Florova G, Reynolds KA (2009). High titer production of tetracenomycins by heterologous expression of the pathway in a *Streptomyces cinnamonensis* industrial monensin producer strain. Metab Eng.

[32] Ikeda H, Ishikawa J, Hanamoto A, Shinose M, Kikuchi H, Shiba T, Sakaki Y, Hattori M, Omura S (2003). Complete genome sequence and comparative analysis of the industrial microorganism *Streptomyces avermitilis*. Nat Biotechnol.

[33] Komatsu M, Uchiyama T, Omura S, Cane DE, Ikeda H (2010). Genome-minimized *Streptomyces* host for the heterologous expression of secondary metabolism. Proc Natl Acad Sci USA.

[34] Komatsu M, Komatsu K, Koiwai H, Yamada Y, Kozone I, Izumikawa M, Hashimoto J, Takagi M, Omura S, Shin-ya K, Cane DE, Ikeda H (2013). Engineered *Streptomyces avermitilis* host for heterologous expression of biosynthetic gene cluster for secondary metabolites. ACS Synth Biol.

[35] Green MR, Sambrook J (2001). Molecular cloning: a laboratory manual.

[36] Takahashi Y, Matsumoto A, Seino A, Ueno J, Iwai Y, Omura S (2002). *Streptomyces avermectinius* sp. nov., an avermectin-producing strain. Int J Syst Evol Microbiol.

[37] Miller GL (1959). Use of dinitrosalicylic acid reagent for determination of reducing sugar. Anal Chem.

