# Supplementary figures and images for: Engineered biosynthesis of milbemycins in the avermectin high-producing strain Streptomyces avermitilis
Source: Microb Cell Fact. 2017 Jan 17;16:9. doi: 10.1186/s12934-017-0626-8 (PMC5240415; doi:10.1186/s12934-017-0626-8)

## Slide 1
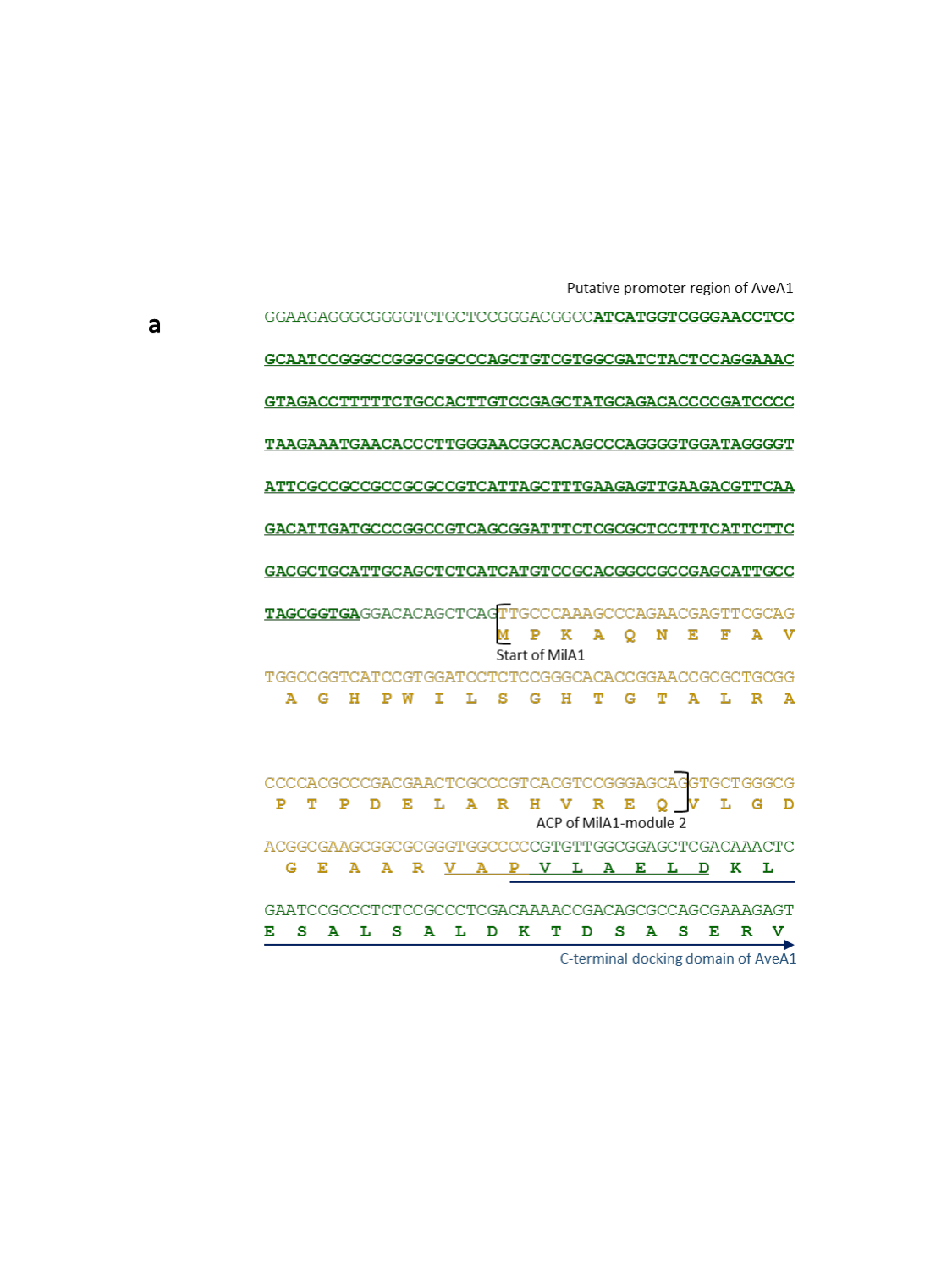

## Slide 2
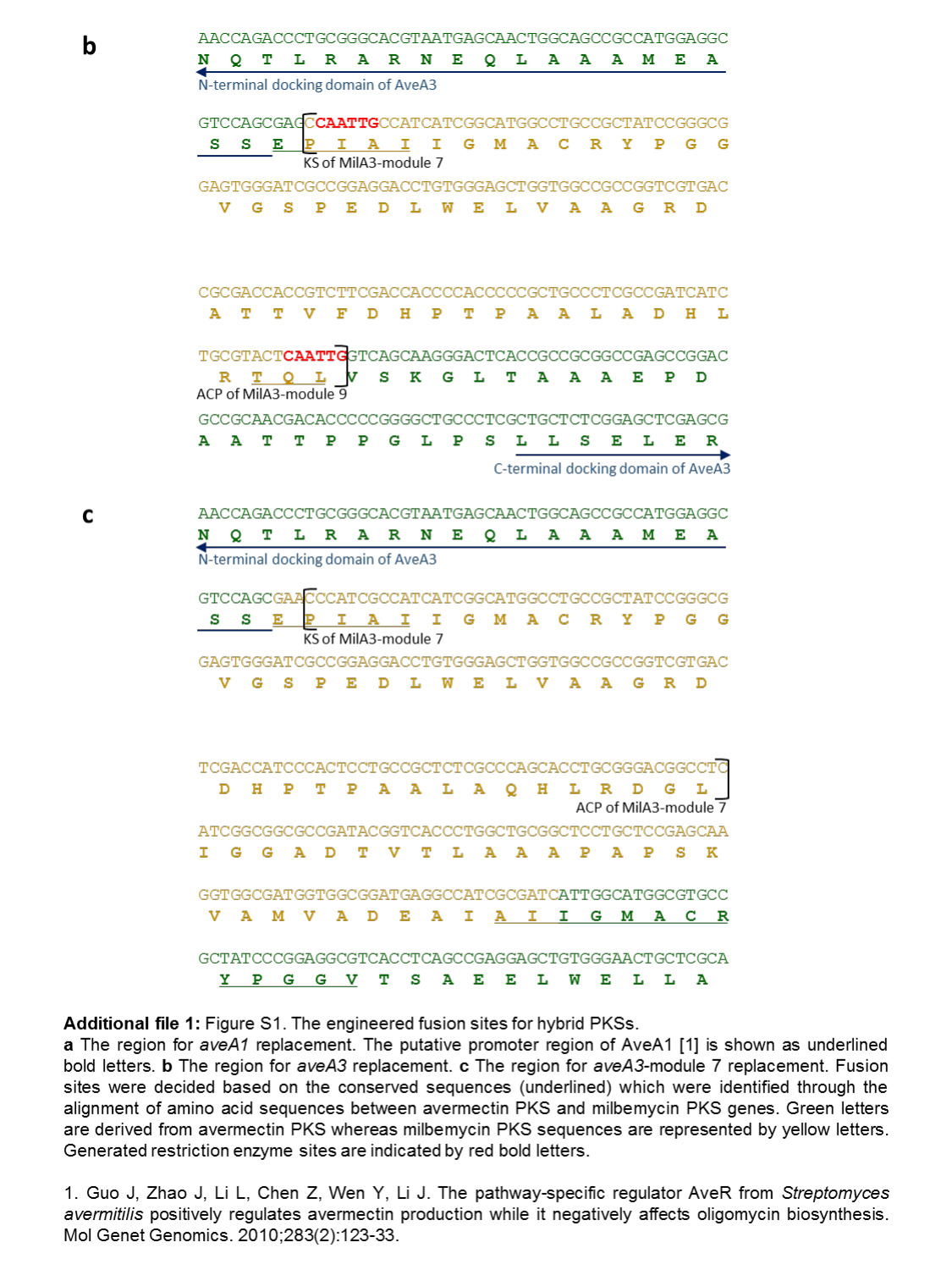

Supplement: Supplementary file 1 — Additional file 1: Figure S1. The engineered fusion sites for hybrid PKSs. [file 12934_2017_626_MOESM1_ESM.pptx]

## Slide 1
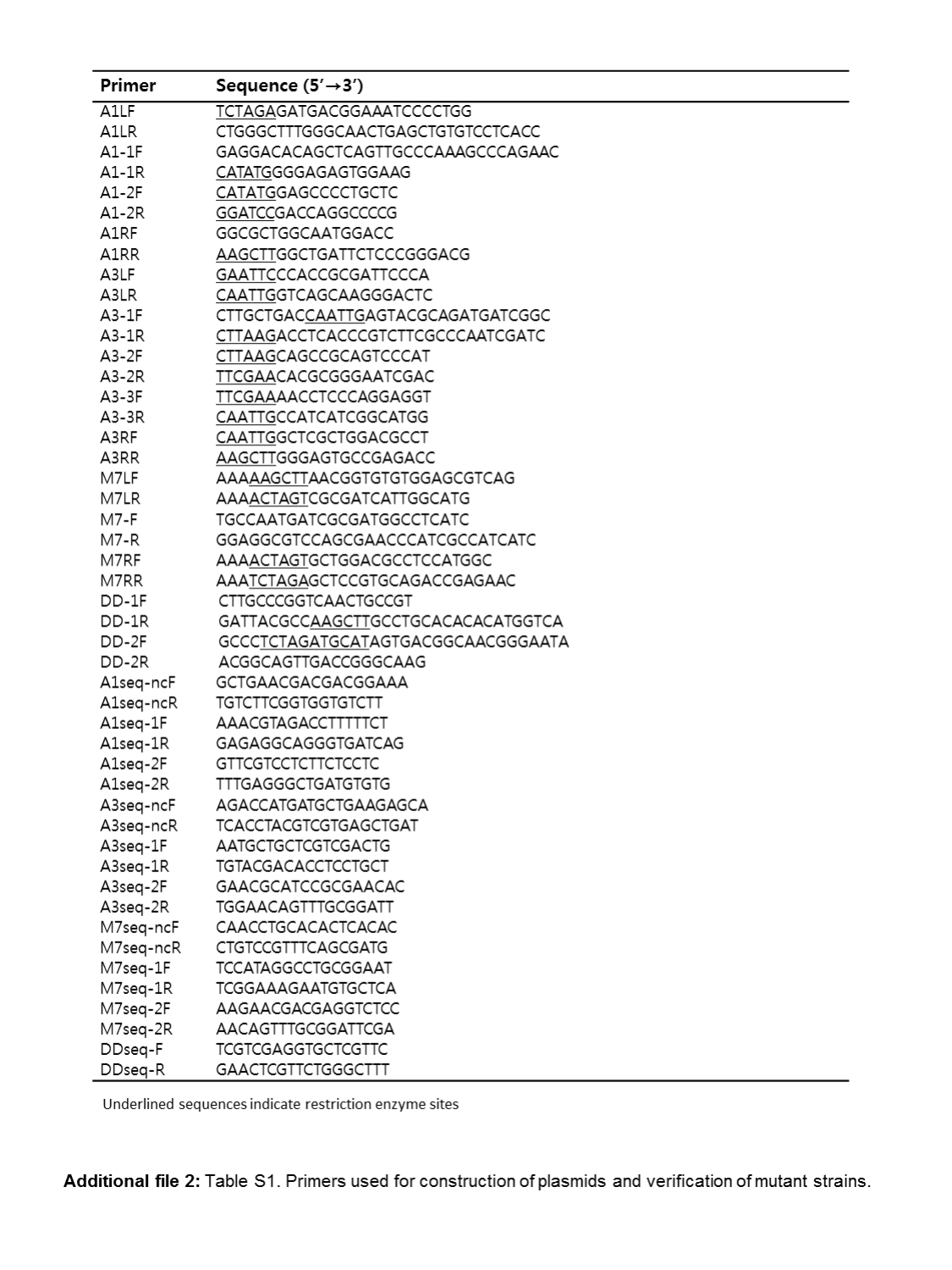

Supplement: Supplementary file 2 — Additional file 2: Table S1. Primers used for construction of plasmids and verification of mutant strains. [file 12934_2017_626_MOESM2_ESM.pptx]

## Slide 1
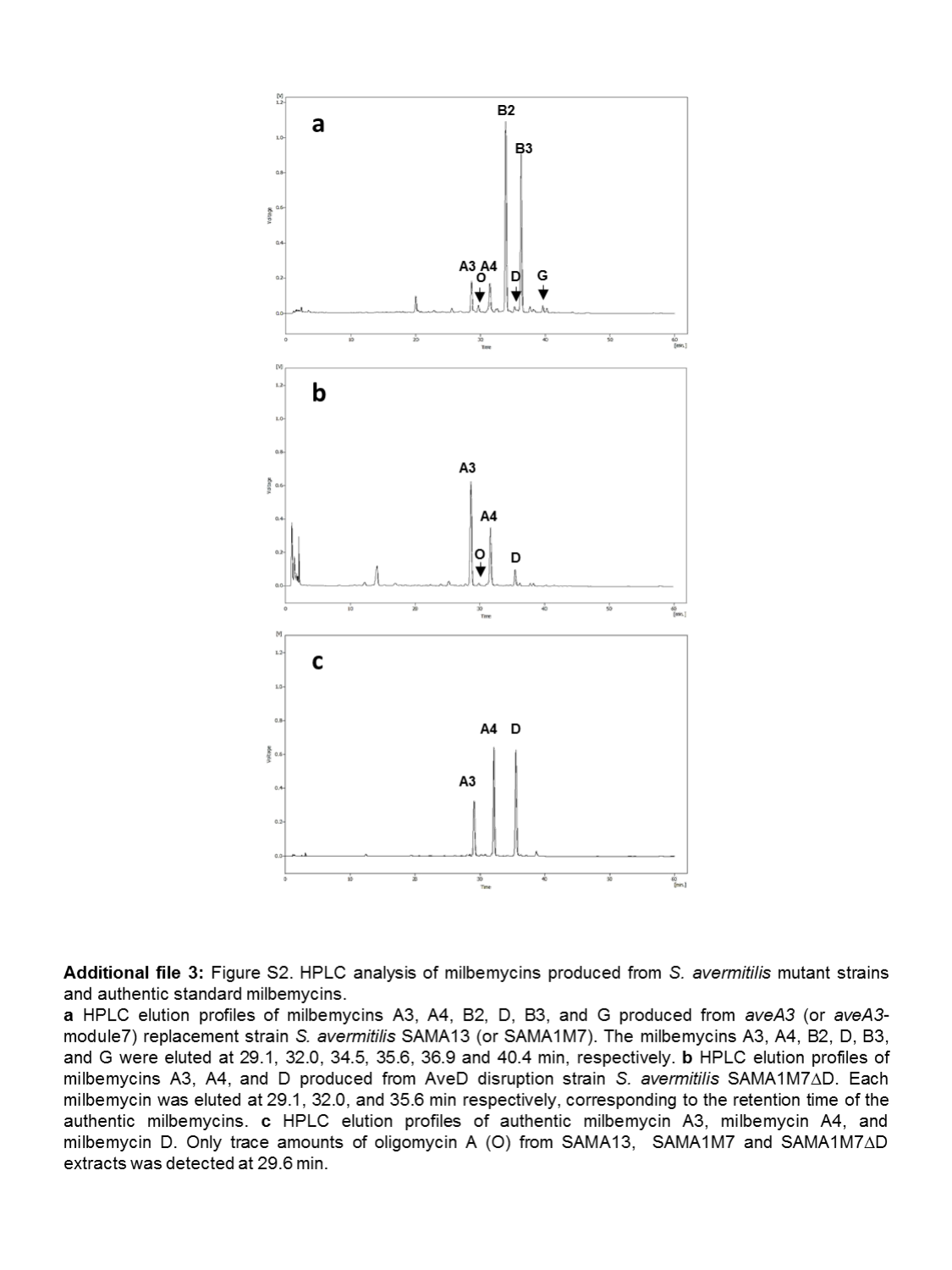

Supplement: Supplementary file 3 — Additional file 3: Figure S2. HPLC analysis of milbemycins produced from S. avermitilis mutant strains and authentic standard milbemycins. [file 12934_2017_626_MOESM3_ESM.pptx]

## Slide 1
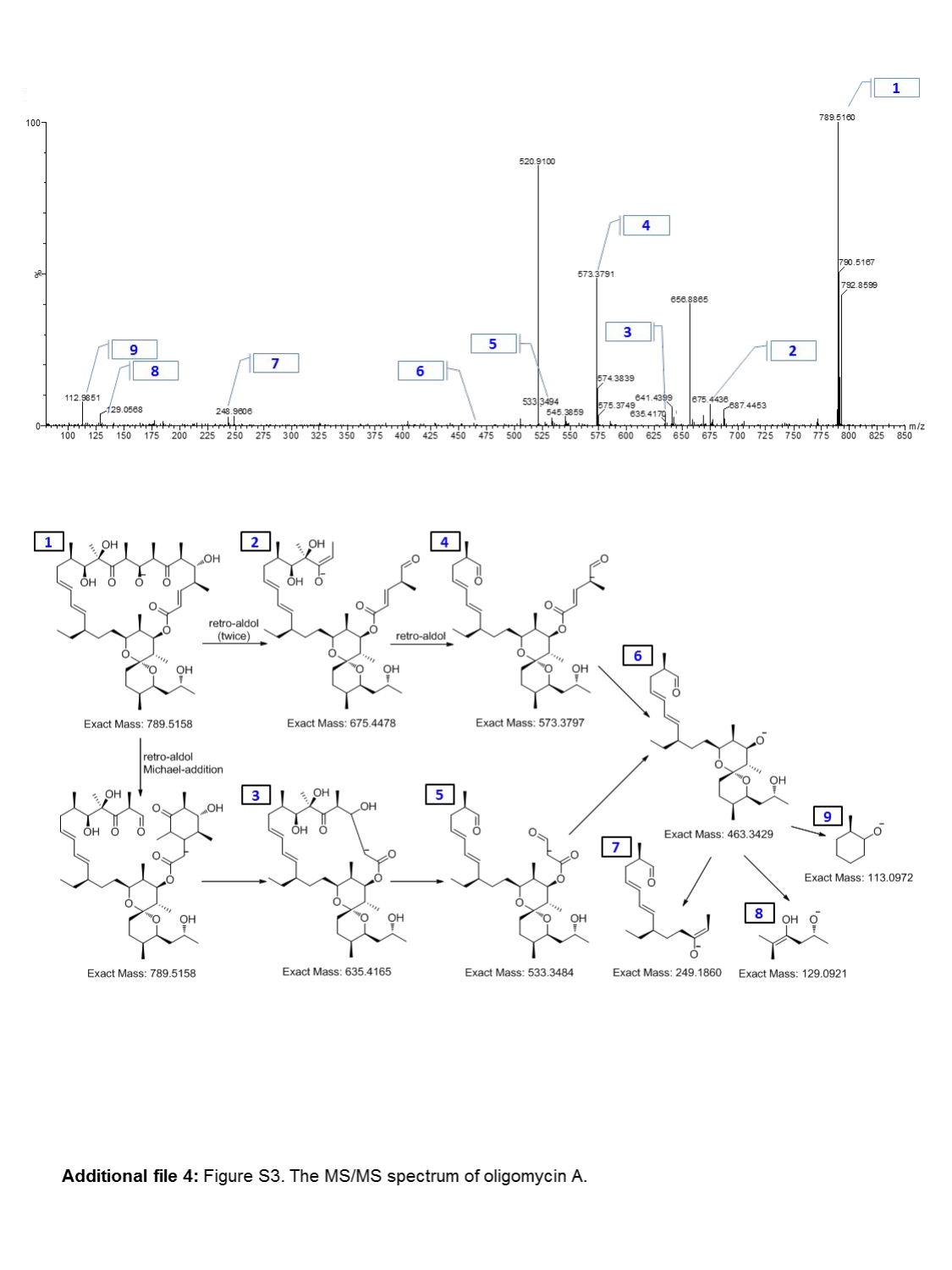

Supplement: Supplementary file 4 — Additional file 4: Figure S3 MS/MS spectrum of oligomycin A produced from S. avermitilis mutant strains. [file 12934_2017_626_MOESM4_ESM.pptx]

## Slide 1
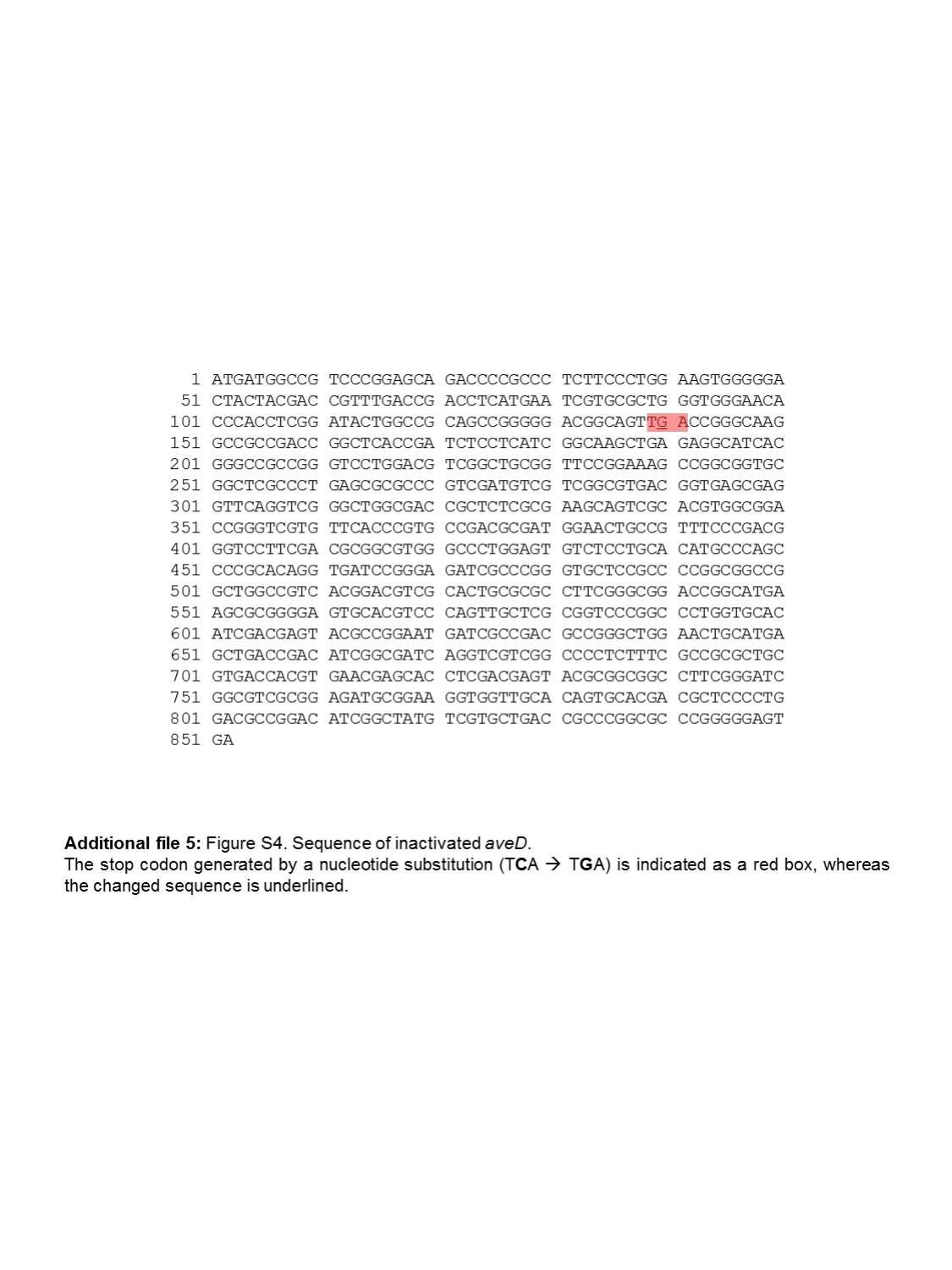

Supplement: Supplementary file 5 — Additional file 5: Figure S4. Sequence of inactivated aveD. [file 12934_2017_626_MOESM5_ESM.pptx]

## Slide 1
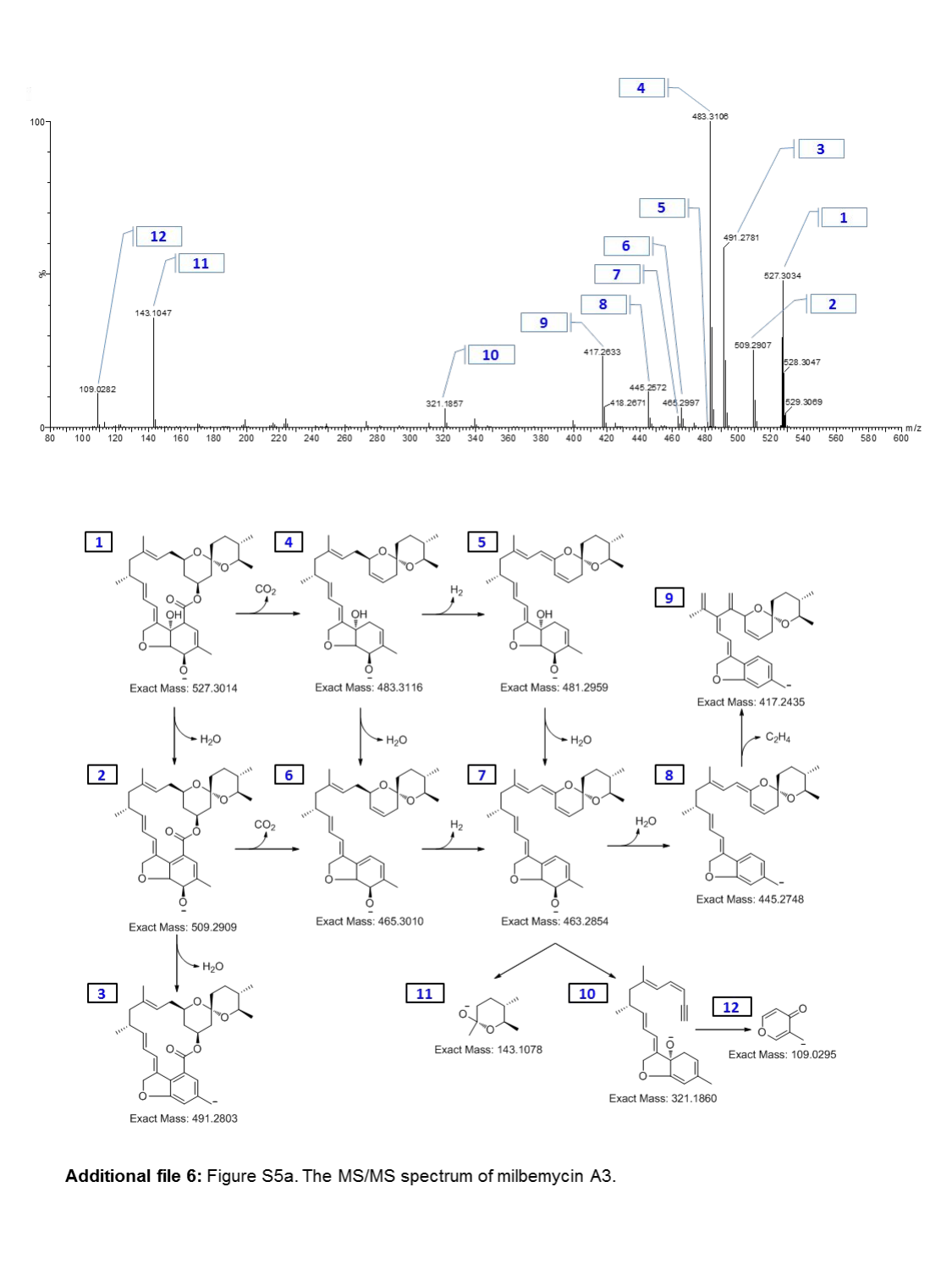

## Slide 2
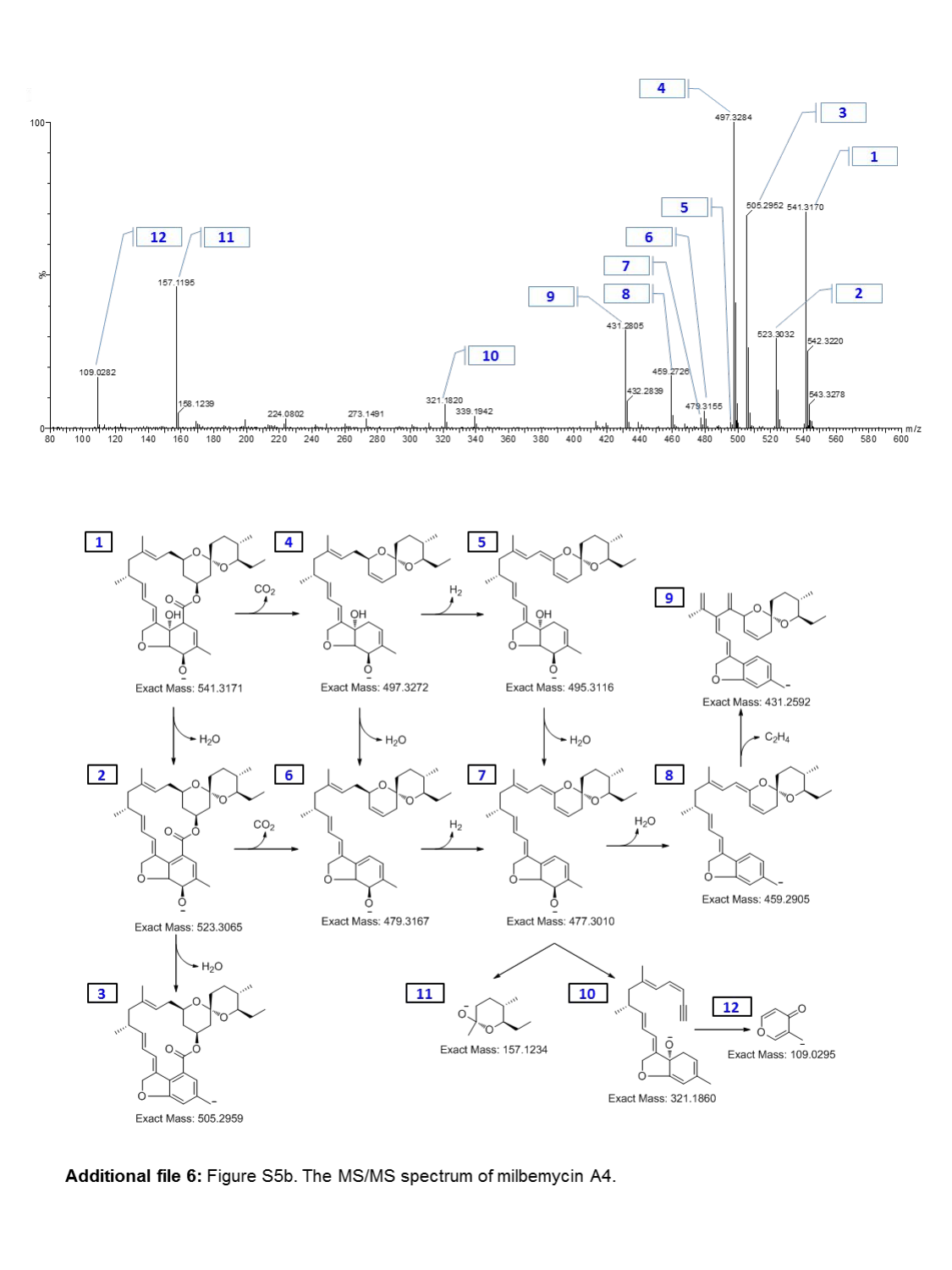

## Slide 3
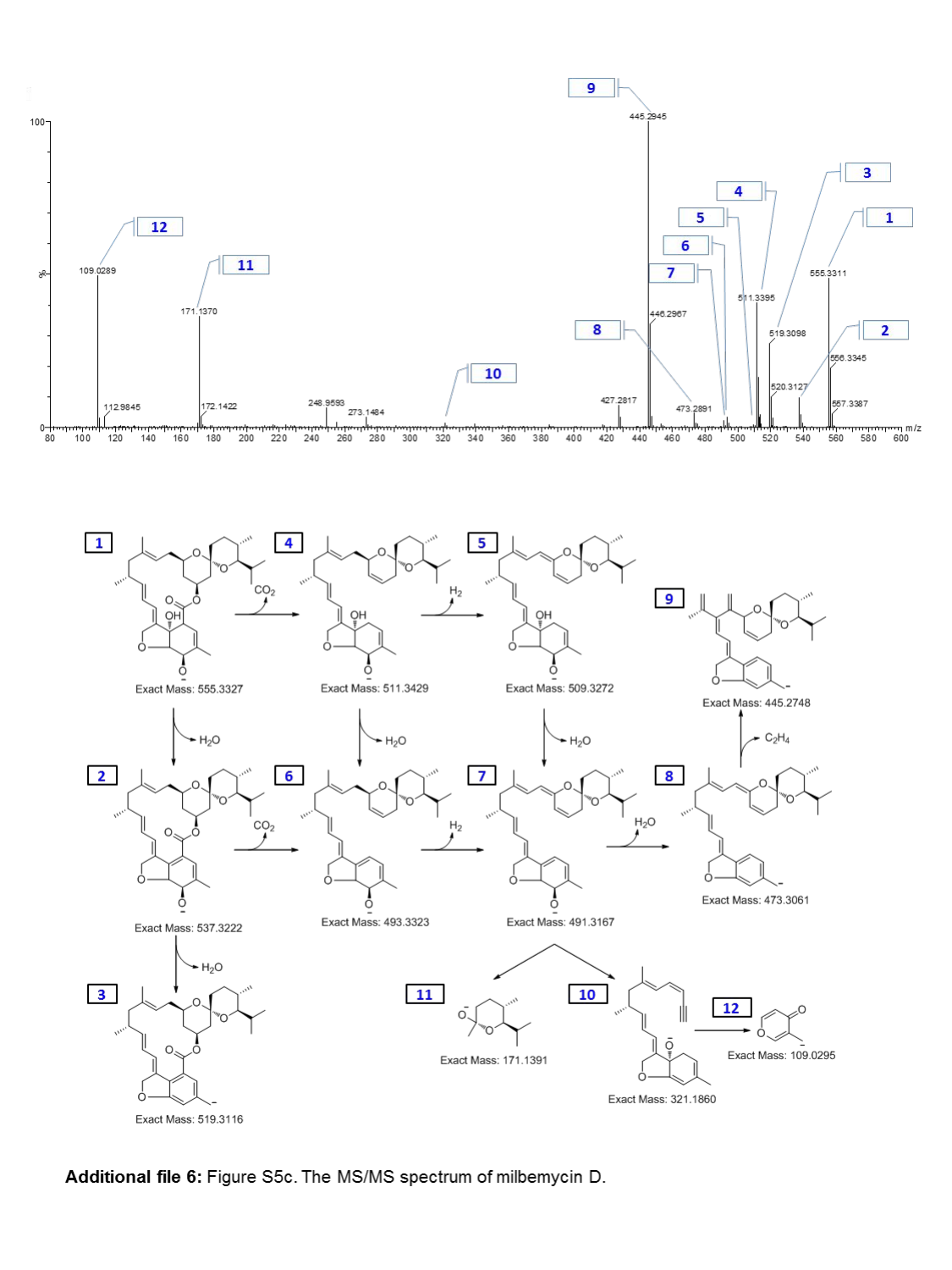

## Slide 4
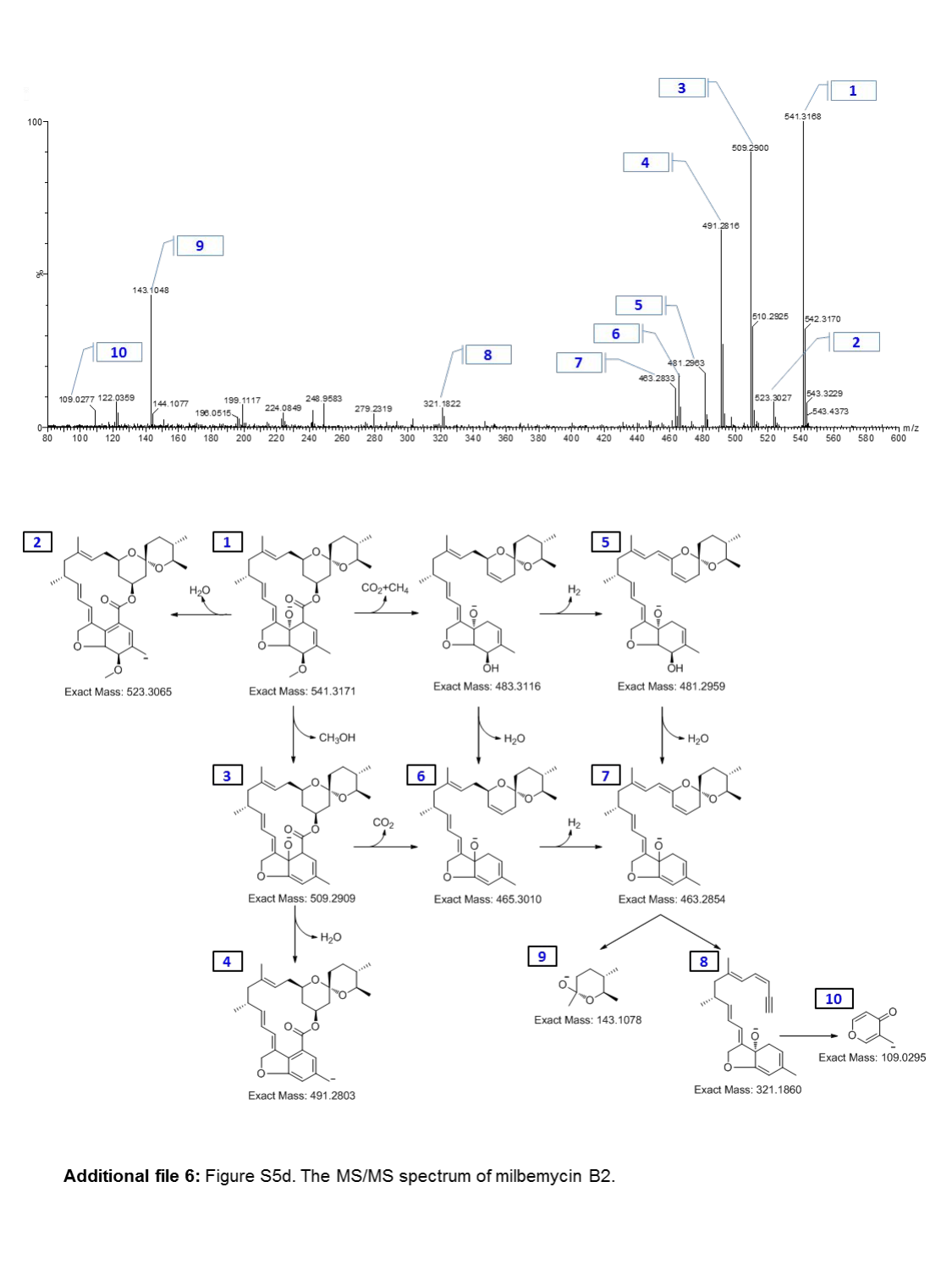

## Slide 5
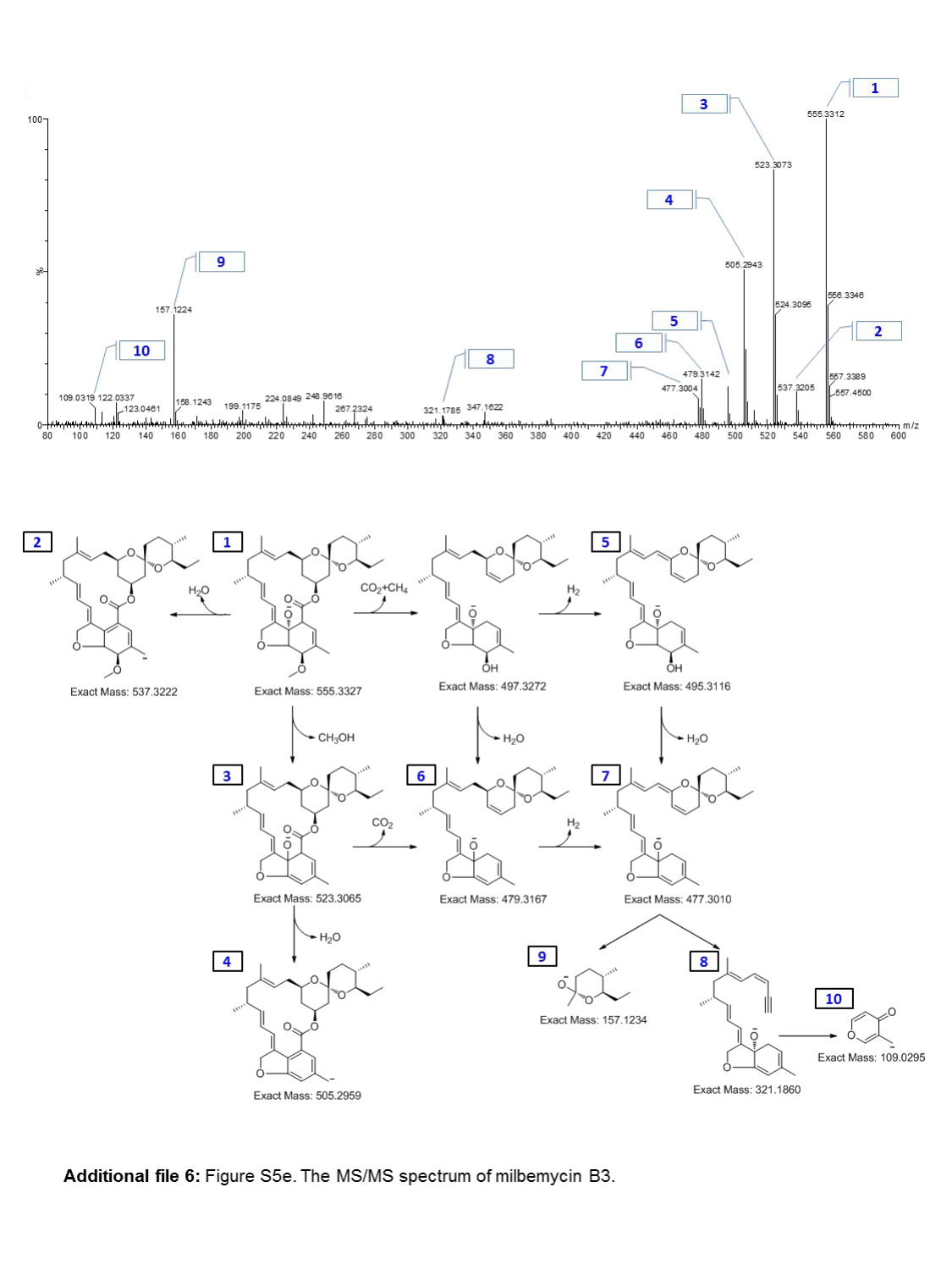

## Slide 6
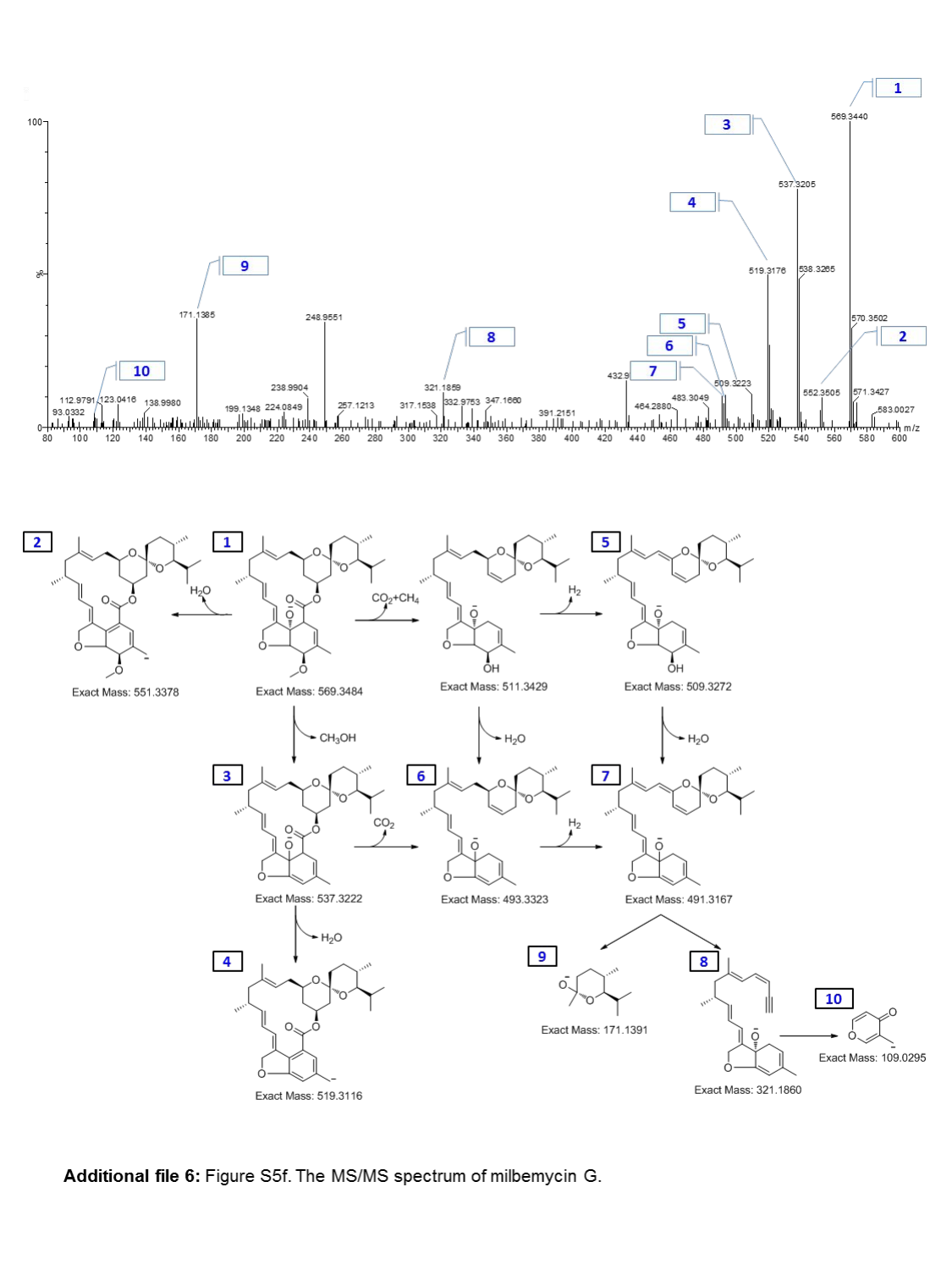

Supplement: Supplementary file 6 — Additional file 6: Figure S5. MS/MS spectra of milbemycins produced from S. avermitilis mutant strains. [file 12934_2017_626_MOESM6_ESM.pptx]
